# Bio-inspired Z-scheme g-C_3_N_4_/Ag_2_CrO_4_ for efficient visible-light photocatalytic hydrogen generation

**DOI:** 10.1038/s41598-018-34287-w

**Published:** 2018-11-07

**Authors:** Yuping Che, Bingxin Lu, Qi Qi, Huaiqiu Chang, Jin Zhai, Kefeng Wang, Zhaoyue Liu

**Affiliations:** 1 Key Laboratory of Bio-inspired Smart Interfacial Science, Technology of Ministry of Education and Beijing Advanced Innovation Center for Biomedical Engineering, Beijing Key Laboratory of Bio-inspired Energy Materials and Devices, School of Chemistry, Beihang University, Beijing, 100191 P. R. China; 2 Henan Key Laboratory of Biomolecular Recognition and Sensing, Shangqiu Normal University, Shangqiu, 476000 P. R. China; 3 National Center for Nanoscience and Technology, Beijing, 100190 P. R. China

## Abstract

Due to low charge separation efficiency and poor stability, it is usually difficult for single-component photocatalysts such as graphitic carbon nitride (g-C_3_N_4_) and silver chromate (Ag_2_CrO_4_) to fulfill photocatalytic hydrogen production efficiently. Z-scheme charge transport mechanism that mimics the photosynthesis in nature is an effective way to solve the above problems. Inspired by photosynthesis, we report Ag_2_CrO_4_ nanoparticles-decorated g-C_3_N_4_ nanosheet as an efficient photocatalyst for hydrogen evolution reaction (HER) with methanol as sacrificial agent. The formation of Z-scheme g-C_3_N_4_/Ag_2_CrO_4_ nanosheets photocatalysts could inhibit the recombination of photogenerated electron-hole pairs, promote the generation of hydrogen by photosplitting of water. The experiment results indicate that g-C_3_N_4_/Ag_2_CrO_4_ nanocomposites present enhanced photocatalytic activity and stability in the H_2_ evolution of water splitting. And the nanocomposites g-C_3_N_4_/Ag_2_CrO_4_(23.1%) show the 14 times HER efficiency compared to that of bare g-C_3_N_4_.

## Introduction

With fossil fuel reserves dwindling every day, there is an urgent need for clean and sustainable alternative energy sources. Hydrogen energy is an attractive alternative resource to fossil fuels due to its high energy density and environmentally friendly characteristics^1–6^. Many methods are used to produce hydrogen, which can be divided into two major categories based on the required raw materials and processes: reforming processes and splitting water processes. The former process is relatively mature and uses widely. However, it has some disadvantages such as high cost, large energy consumption, low efficiency, and complicated equipments and processes. Splitting water processes contains electrolytic water splitting, photocatalytic water splitting, bio-photocatalytic water splitting and thermochemical water splitting. Water electrolysis technology is relatively mature, with simple equipment, no pollution, and high product purity but large energy consumption; water biophotolysis process Bio-photocatalytic water splitting requires harsh reaction environments but has poor stability. There is no problem in the feasibility and high efficiency for thermochemical water splitting technology, but further research is needed to reduce costs and achieving efficient recycling. Among them, photocatalytic water splitting is a more efficient pathway to produce hydrogen gas and also does not generate any undesirable byproducts^7–17^. Since the discovery of the Honda-Fujishima effect in 1972^18^, photocatalytic hydrogen production through water splitting has become a promising technology for utilizing renewable solar energy^19–24^. Development of efficient photocatalytic systems for hydrogen evolution via photoinduced water splitting is an active field of energy research. However, due to narrow absorption range, poor stability, low charge-separation efficiency and weak redox ability, it is often difficult for a single-component photocatalyst to fulfill this requirement. The heterostructure composed of two semiconductor catalysts can increase the catalytic efficiency^25–29^. Photosynthesis as a nature heterostructure system is widely studied due to its fast and efficient photocatalytic reaction.

**Figure 1:**
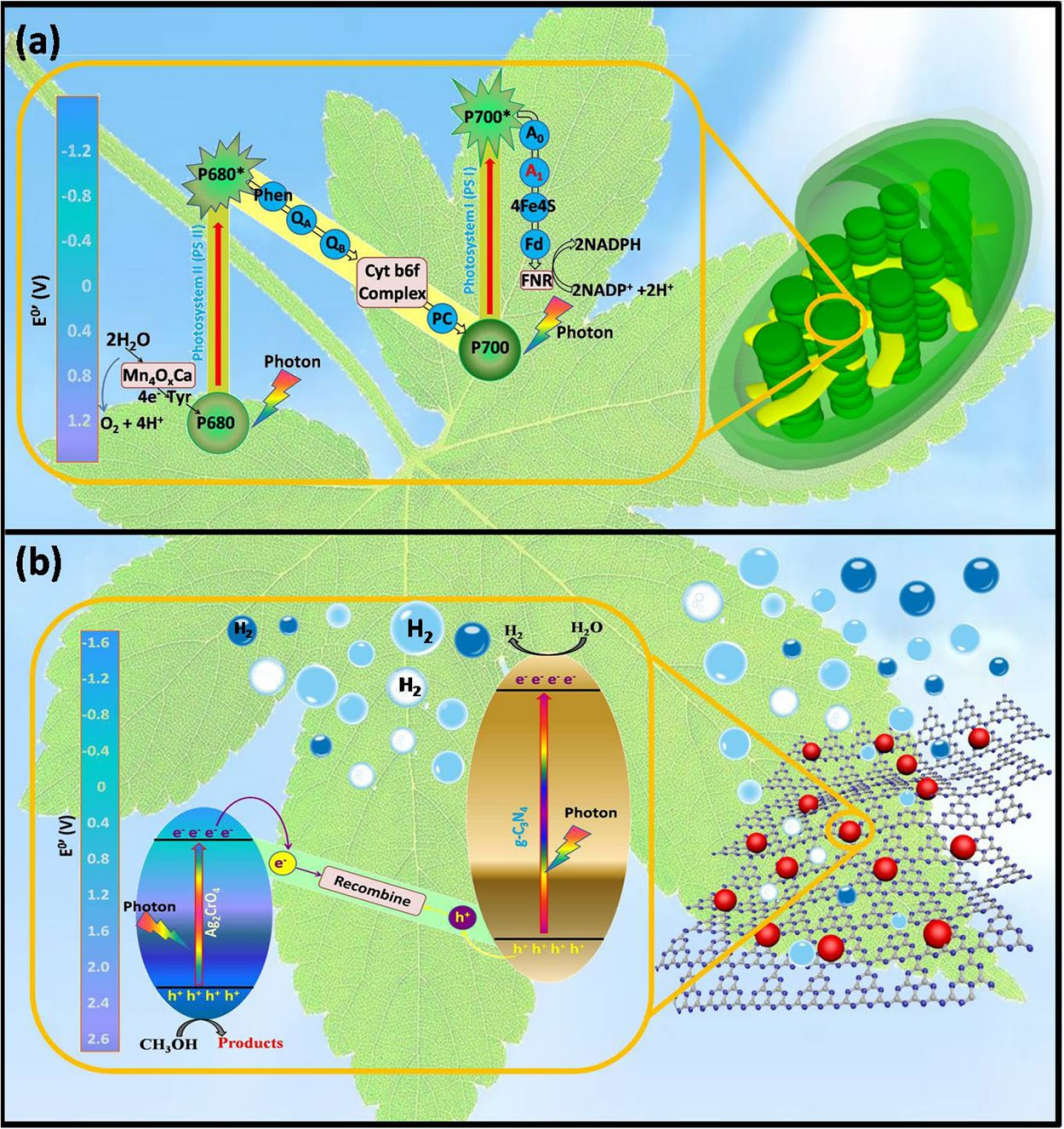
(**a**) Natural photosynthesis (the electrons in HOMO of PS II are excited to its LUMO under solar light; Then, the photogenerated electrons in LUMO of PS II are transferred to HOMO of PS I through the electron mediator. Further, the electrons in HOMO of PS I are excited to its LUMO. The transfer process of electrons in the figure constitutes a shape of letter Z, so it is called a Z-scheme) and (**b**) an artificial analogy composed of an organic semiconductor g-C_3_N_4_ (equivalent to PS I) and silver chromate (equivalent to PS II) (the electrons in HOMO of Ag_2_CrO_4_ are excited to its LUMO under visible light irradiation; Then, the electrons in HOMO of g-C_3_N_4_ are excited to its LUMO under visible light irradiation; the excited electrons of Ag_2_CrO_4_ and the holes of g-C_3_N_4_ recombine; as a result, the migration route of electrons is a Z-scheme).

In natural photosynthesis process, the Z-scheme photoreaction system is an important part of the plant photosynthesis, which involves two photochemical reactions and a series of intermediate enzymatic redox reactions. The electron transfer process is shown in Fig. 1a. After a series of reactions, the photogenerated electrons with high reducing ability and holes with high oxidizing ability are left in the LUMO of PS I and the HOMO of PS II, respectively. The transfer process of electrons in the figure constitutes a shape of letter Z, so it is called a Z-scheme^30–32^. This system was first proposed by Bard in 1979 after studying the photosynthesis of plants^33^. The Z-scheme photocatalytic system shows excellent redox ability in due to the different band gap of two semiconductor with broadening light absorption and the special charge transfer path ensuring high separation efficiency of photo-generated charges. Considering these unique advantages of the natural Z-scheme photocatalytic system, the artificial Z-scheme photocatalytic systems have been developed and used widely in photocatalytic field^25,34–38^. The artificial heterogeneous Z-scheme photocatalytic systems, mimicking the natural photosynthesis process, overcome the drawbacks of single-component photocatalysts and satisfy those aforementioned requirements.

For the first time, Martin^39^ reported that the robust organic semiconductor g-C_3_N_4_ can be integrated into a Z-scheme water splitting in a liquid system. While in this system, they used an electron acceptor/donor (NaI/IO^3−^) pair which they all absorb light to the different extent and occur back reactions. Therefore, the number of adsorbed photons by photocatalysts decreases and the effective number of photogenerated electrons and holes decrease sharply^30^. Mimicking the natural photosynthesis process, some all solid-state Z-scheme photocatalyst system based on g-C_3_N_4_ with other semiconductors were obtained and used, such as WO_3_, Ag_3_PO_4_, BiOX (X=Cl, Br, I), V_2_O_5_, etc^40–44^. A Z-scheme system of g-C_3_N_4_/WO_3_ was used to generate hydrogen and its separation mechanisms were also studied deeply^40,45,46^. He *et al*.^41^ reported that the Z-Scheme Ag_3_PO_4_/g-C_3_N_4_ composite was used to convert CO_2_ to fuel such as CO, methanol, methane and ethanol. Liu *et al*.^44^ synthetized a ternary composite photocatalyst g-C_3_N_4_/Ag_3_PO_4_/Ag_2_MoO_4_ for water splitting. BiOCl and BiOI were also compounded with g-C_3_N_4_ as high efficiency photocatalysts^43,47^. As a promising catalyst, Bi_2_WO_6_ was also combined with g-C_3_N_4_ to form a Z-scheme catalytic system^48^.

Silver chromate (Ag_2_CrO_4_) as a narrow band gap semiconductor with band gap of 1.80 eV has unique electronic structure, crystal structure^49^, excellent light sensitivity and high photocatalytic activity^50,51^. Hence, in recent years, some Ag_2_CrO_4_-based photocatalysts have been fabricated^51–53^. However, similar to other silver-based photocatalysts, the big aggregated particle size and easy photocorrosion properties seriously caused poor stability and restricted the photocatalytic performance of the Ag_2_CrO_4_ photocatalyst. The g-C_3_N_4_/Ag_2_CrO_4_ nanocomposites were synthesized and characterized, the dispersion and light stability of Ag_2_CrO_4_ were improved greatly. And their catalytic hydrogen evolution activity was evaluated when using methanol as the sacrificial electron donor under visible light. The as-obtained g-C_3_N_4_/Ag_2_CrO_4_ composites showed distinctly enhanced photo-catalytic activity than that of pure g-C_3_N_4_ nanosheets on the evolution of H_2_ under visible light irradiation. Compared to the two individuals, the enhancement of HER efficiency was mainly ascribed to the synergetic effect between Ag_2_CrO_4_ and g-C_3_N_4_ (as shown in Fig. 1b), effectively improved photogenerated electron-hole pairs separation efficiency via the direct Z-scheme charge transfer mechanism. Notably, the photocorrosion of Ag_2_CrO_4_ was efficiently hindered due to this synergistic effect. Furthermore, a possible direct Z-scheme mechanism for the enhanced photocatalytic activity of the g-C_3_N_4_/Ag_2_CrO_4_ composite was also discussed based on the relative band gap positions of these two semiconductors. The existence of the Z-scheme mechanism means strong redox ability and high transfer efficiency of photogenerated electron-hole pairs. This research will broaden the studies of Ag_2_CrO_4_ and g-C_3_N_4_ photocatalysts with excellent photocorrosion inhibition ability and high photocatalytic activity under visible light.

## Results and Discussion

### Phase structure and morphology analysis

**Figure 2:**
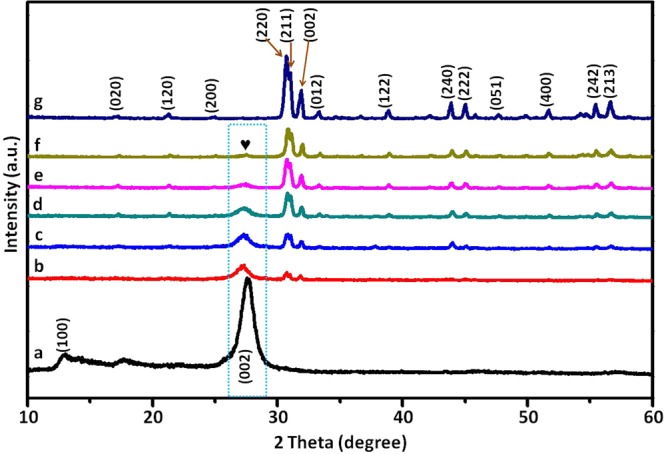
XRD patterns of (**a**) g-C_3_N_4_, (**b**) g-C_3_N_4_/Ag_2_CrO_4_(9.1%), (**c**) g-C_3_N_4_/Ag_2_CrO_4_(16.7%), (**d**) g-C_3_N_4_/Ag_2_CrO_4_(23.1%), (**e**) g-C_3_N_4_/Ag_2_CrO_4_(28.6%), (**f**) g-C_3_N_4_/Ag_2_CrO_4_(33.3%) and (**g**) Ag_2_CrO_4_.

In order to analyze the crystal structure of the prepared g-C_3_N_4_, g-C_3_N_4_/Ag_2_CrO_4_ composites, and Ag_2_CrO_4_, the XRD patterns were obtained (as shown in Fig. 2). Fig. 2a was the pattern of g-C_3_N_4_, two diffraction peaks at 27.6° and 13.0° were indexed to (002) and (100) planes of hexagonal g-C_3_N_4_ (JCPDS card No. 87–1526), corresponding to the graphite-like stacking and the in-plane structural repeating motifs of the conjugated aromatic units of g-C_3_N_4_^54^. From Fig. 2b–f, the patterns correspond to the samples with an increasing Ag_2_CrO_4_ mass ratio to g-C_3_N_4_. For the g-C_3_N_4_/Ag_2_CrO_4_ composites, orthorhombic phase Ag_2_CrO_4_ and hexagonal phase g-C_3_N_4_ were both observed and no other impurity peaks were found, implying that g-C_3_N_4_ and Ag_2_CrO_4_ keep pure phase and no impurities formed in g-C_3_N_4_/Ag_2_CrO_4_ composites. Moreover, the peaks of Ag_2_CrO_4_ in g-C_3_N_4_/Ag_2_CrO_4_ composites were becoming clearer with the increasing of Ag_2_CrO_4_. And at the same time the peaks of g-C_3_N_4_ gradually decreased. Fig. 2g was the pattern of Ag_2_CrO_4_. It was clear to see that all diffraction peaks of the as-prepared Ag_2_CrO_4_ coincided well with the orthorhombic phase of Ag_2_CrO_4_ (JCPDS No. 26-0952).

**Figure 3:**
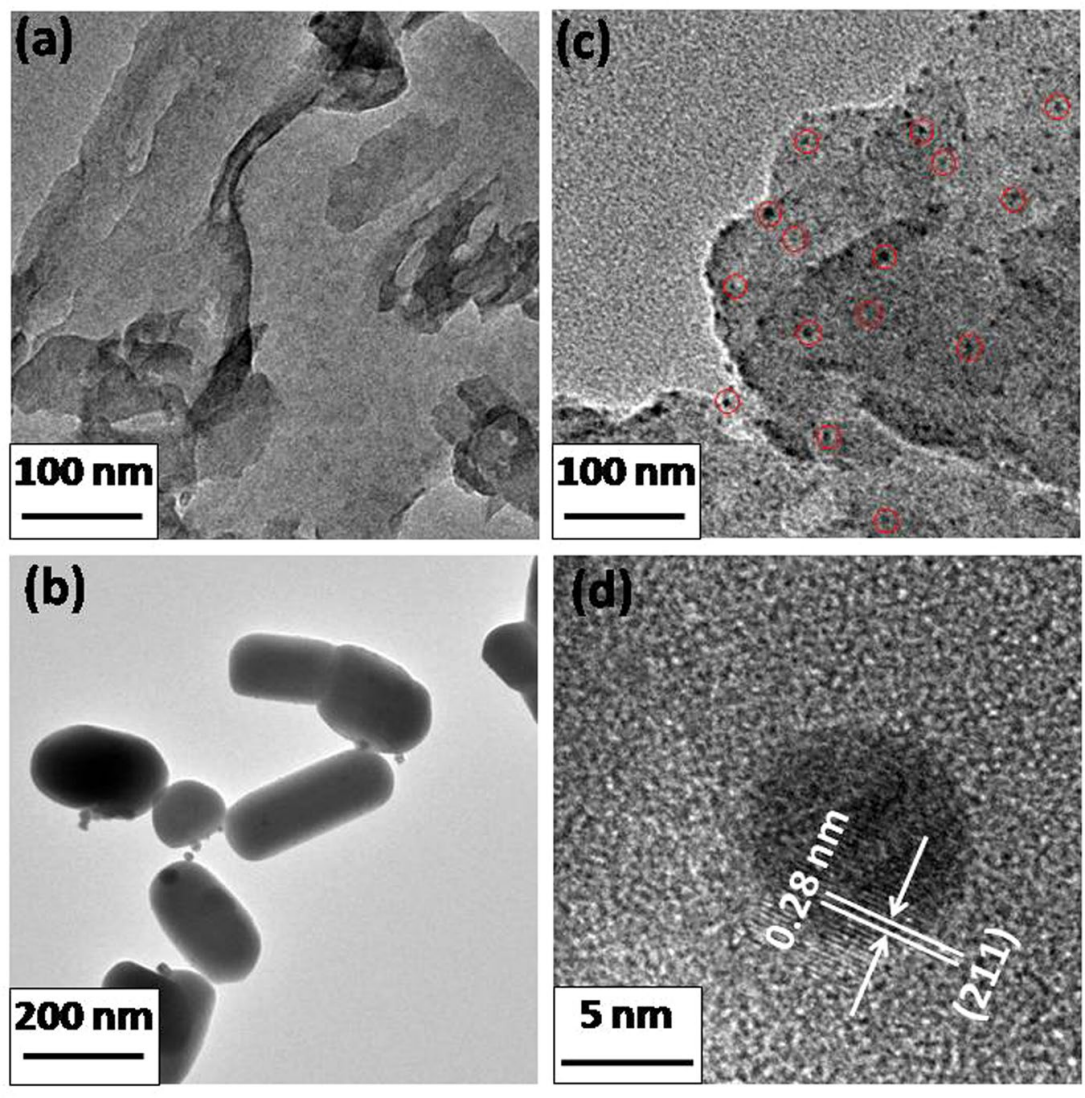
TEM images of (**a**) g-C_3_N_4_, (**b**) Ag_2_CrO_4_, (**c**) g-C_3_N_4_/Ag_2_CrO_4_(23.1%) (Ag_2_CrO_4_ were labeled by red circles), (**d**) HRTEM of g-C_3_N_4_/Ag_2_CrO_4_(23.1%).

In order to observe the morphology of the bare g-C_3_N_4_, Ag_2_CrO_4_ and the as-prepared g-C_3_N_4_/Ag_2_CrO_4_ composite, TEM was performed (see Fig. 3). The pure g-C_3_N_4_ sample possessed a very thin 2D layer structure (shown as Fig. 3a), which meant that the g-C_3_N_4_ was prepared successfully. As to the pristine Ag_2_CrO_4_ sample (shown in Fig. 3b), besides the obvious aggregations there was no special morphological features, and the sizes of Ag_2_CrO_4_ particles were between 140–300 nm and asymmetrical. The TEM image of the g-C_3_N_4_/Ag_2_CrO_4_ composite with the mass weight ratio 23.1% was showed in Fig. 3c. From the TEM picture, we could see that the Ag_2_CrO_4_ particles (labeled by red circles) with the size below 10 nm were uniformly embedded on g-C_3_N_4_. The adding of graphene-like g-C_3_N_4_ nanosheets made the Ag_2_CrO_4_ particles composites in the g-C_3_N_4_/Ag_2_CrO_4_(23.1%) closely enwrapped by g-C_3_N_4_ and much smaller than pure Ag_2_CrO_4_ nanoparticles. These results indicate that g-C_3_N_4_ nanosheets could not only serve as a graphene-like substrate to attach Ag_2_CrO_4_ nanoparticles on their surface, but also inhibit Ag_2_CrO_4_ nanoparticles aggregating into the big particles. Therefore, g-C_3_N_4_/Ag_2_CrO_4_ composite photocatalyst with closely interconnections between g-C_3_N_4_ and Ag_2_CrO_4_ nanoparticles were successfully synthesized, which would be beneficial for the transfer of the photogenerated electron-hole pairs. In Fig. 3d, obviously, the clear fringes spacing was ca. 0.28 nm, corresponding to the (211) lattice plane of orthorhombic phase Ag_2_CrO_4_^55,56^. The composites with other different weight ratios (9.1%, 16.7%, 28.6%, and 33.3%) were shown in Fig. S1. We could see that when the ratio of the Ag_2_CrO_4_ below 23.1%, small Ag_2_CrO_4_ nanoparticles (below 10 nm) were uniformly embedded on g-C_3_N_4_ (labeled by red circles in Figs 3b, S1a and S1b). While the theoretical weight ratio was increased to 28.6%, Ag_2_CrO_4_ began to aggregate, the size of Ag_2_CrO_4_ grow to above 20 nm. When the theoretical ratio was 33.3%, the aggregation was very serious just like the pure Ag_2_CrO_4_.

Based on Figs 3 and S1, a possible formation mechanism of g-C_3_N_4_/Ag_2_CrO_4_ composites is proposed. Obviously, the structure of Ag_2_CrO_4_ ranged from nanoparticles (below 10 nm) to sub-microparticles (140–300 nm) with the decreasing of g-C_3_N_4_ mass fraction, which strongly confirmed that the g-C_3_N_4_ sheets played vital role in the formation of the nanojunction. Many investigations showed that large specific surface area and two-dimensional structure of g-C_3_N_4_ could provide a large scaffold for anchoring various substrates^57,58^. In addition, the surface of urea-derived graphitic g-C_3_N_4_ possessed positive charge with abundant alamino groups (C-NH_x_), which could provide a suitable environment for attracting negative charge particles via electrostatic attraction^59,60^. Accordingly, in our study, the CrO_4_^2−^ ions could adsorb onto g-C_3_N_4_ sheets via the electrostatic force in g-C_3_N_4_ suspension. The anchored CrO_4_^2−^ ions would form Ag_2_CrO_4_ nanocrystals *in situ* on the surface of g-C_3_N_4_ sheets during the reaction, then the tiny nanocrystal nucleus grows into the nanoparticles through oriented growth on the surface of g-C_3_N_4_ support^61^. Eventually, the Ag_2_CrO_4_ nanoparticles uniformly and tightly distribute onto the surface of g-C_3_N_4_ sheets (As shown in Figs 3c and S1a,b). However, with the increase of Ag_2_CrO_4_ mass ratio, the capacity of electrostatic attraction between g-C_3_N_4_ and CrO_4_^2−^ ions was decreased because the effective positive charge surface of g-C_3_N_4_ was decreasing. When the mass fraction of Ag_2_CrO_4_ was more than 28.6%, the excess of CrO_4_^2−^−ions could not tightly adsorb on the surface of g-C_3_N_4_ sheets and would grow into Ag_2_CrO_4_ nanoparticles freely during the reaction progress. However, these free Ag_2_CrO_4_ nanocrystals possessed high specific surface energy, which would assemble spontaneously and form hierarchical sub-microparticles for reducing the interfacial energy^52,62^. As a result, part of Ag_2_CrO_4_ nanoparticles uniformly grew on the surface of g-C_3_N_4_ due to the strong electrostatic attraction between CrO_4_^2−^− ions and C-NH_x_, whereas other Ag_2_CrO_4_ nanoparticles were densely self-assembled and formed 3D hierarchical structures which covered the g-C_3_N_4_ sheets (Fig. S1c). With the mass fraction of free Ag_2_CrO_4_ nanoparticles further increasing, the crystal growth of nanoparticles would cause the exfoliation of the g-C_3_N_4_ sheets (Fig. S1d)^63,64^. The Ag_2_CrO_4_ sub-microparticles could directly contacted with g-C_3_N_4_ sheets, which were shown in Fig. S1d. The pure Ag_2_CrO_4_ 3D hierarchical structures was obtained with the absence of g-C_3_N_4_. It revealed that when the particles of Ag_2_CrO_4_ nanoparticles were not restricted by the functional alamino groups on the surface of g-C_3_N_4_ sheets, they would assemble spontaneously in a random way to form 3D sub-microparticles (shown as Fig. 3c)^49^. All in all, the results clearly confirmed that the mass ratio of g-C_3_N_4_ could be a key parameter for formation of g-C_3_N_4_/Ag_2_CrO_4_ nanocomposites. The positive charge and 2D structure of g-C_3_N_4_ sheets could provide a suitable environment for the growth of nanoparticles.

### Component analysis

**Figure 4:**
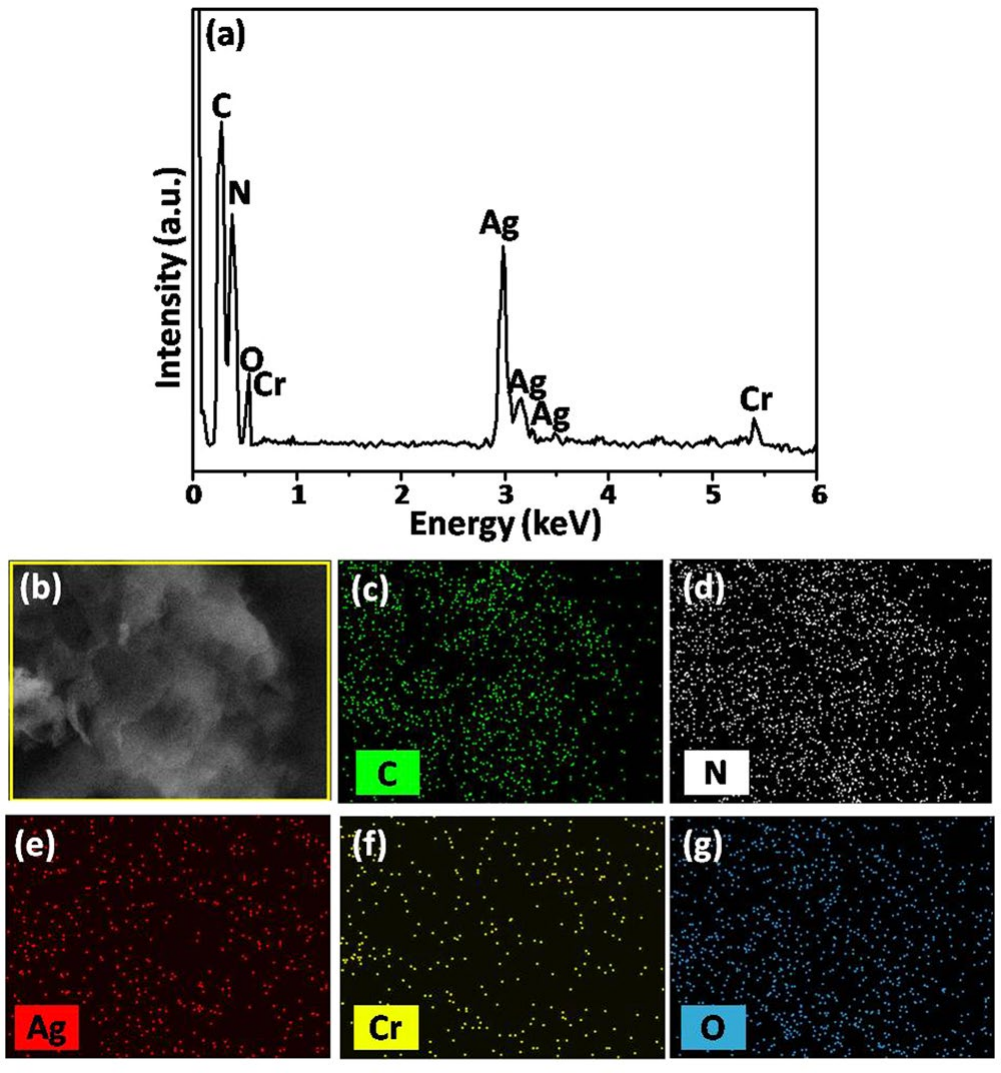
(**a**) EDS spectra for the g-C_3_N_4_/Ag_2_CrO_4_(23.1%), (**b**) SEM of g-C_3_N_4_/Ag_2_CrO_4_(23.1%), (**c**–**g**) EDS mapping for different elements of the g-C_3_N_4_/Ag_2_CrO_4_(23.1%) nanocomposite.

The elemental compositions of g-C_3_N_4_/Ag_2_CrO_4_(23.1%) nanocomposite were explored by EDS technique and the results were shown in Fig. 4. It could be seen that the g-C_3_N_4_/Ag_2_CrO_4_(23.1%) composite was composed of only C, N, Ag, Cr and O elements and no other element or impurity found, demonstrating that the existence of g-C_3_N_4_ and Ag_2_CrO_4_ in the as-fabricated g-C_3_N_4_/Ag_2_CrO_4_(23.1%) composite (shown as in Fig. 4a). Quantitative analysis of the g-C_3_N_4_/Ag_2_CrO_4_(23.1%) nanocomposite showed that weight percents of C, N, Ag, Cr and O elements were 29.4, 47.2, 15.0, 3.6, and 4.8%, respectively, which they were close to the theoretical percents of 30.1, 46.8, 15.0, 3.6 and 4.5%, respectively. To further investigate elemental composition and distribution uniformity, the elemental maps for g-C_3_N_4_/Ag_2_CrO_4_(23.1%) composite were displayed in Fig. 4c–g, which indicated that Ag, Cr and O elements were homogeneously distributed in the whole host of the g-C_3_N_4_/Ag_2_CrO_4_(23.1%) composite. Considering the Ag, Cr and O presented in the form of Ag_2_CrO_4_, these analyses demonstrated Ag_2_CrO_4_ tended to integrate with g-C_3_N_4_ nanosheets firmly and then formed hybrid structures. Except that, X-ray photoelectron spectroscopy and Fourier-transform infrared spectroscopy were also used to analyze the composition of nanomaterials (as shown in Figs S2 and S4).

### Optical properties of the g-C_3_N_4_/Ag_2_CrO_4_ composites

**Figure 5:**
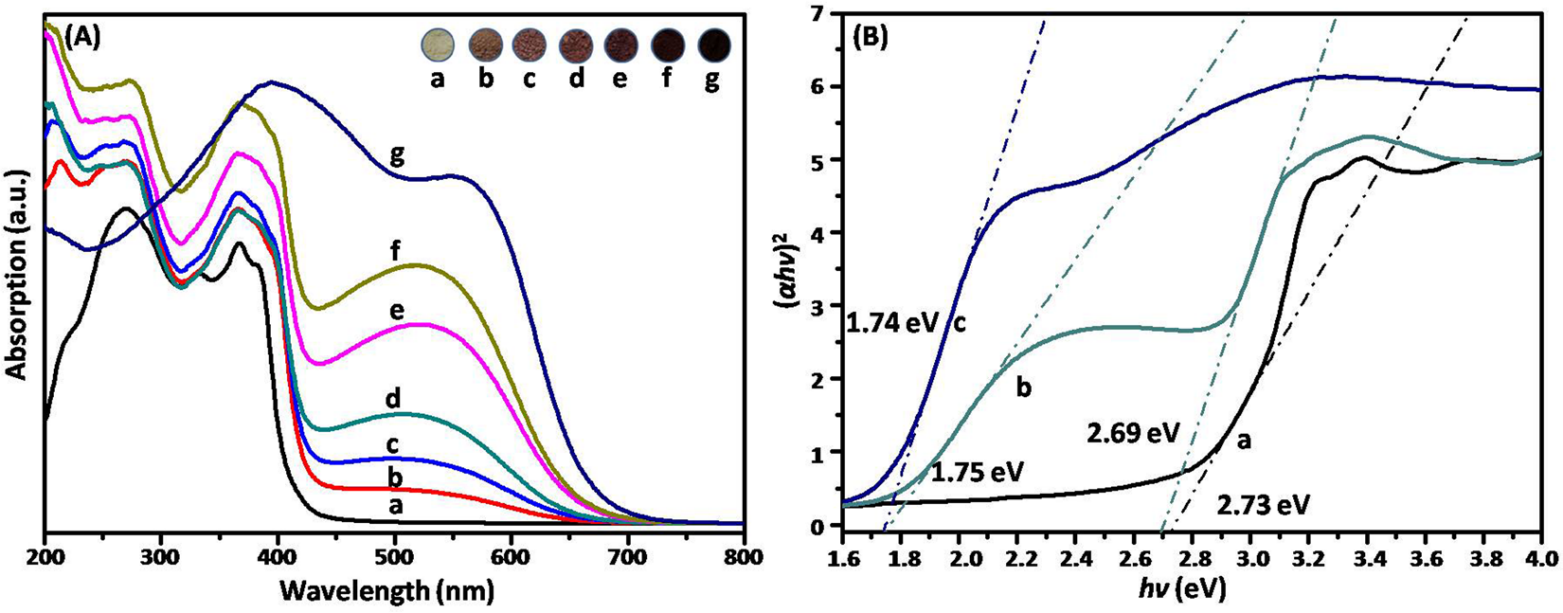
(**A**) UV–vis DRS (insert are the photos of samples) of (a) g-C_3_N_4_, (b) g-C_3_N_4_/Ag_2_CrO_4_(9.1%), (c) g-C_3_N_4_/Ag_2_CrO_4_(16.7%), (d) g-C_3_N_4_/Ag_2_CrO_4_(23.1%), (e) g-C_3_N_4_/Ag_2_CrO_4_(28.6%), (f) g-C_3_N_4_/Ag_2_CrO_4_(33.%) and (g) Ag_2_CrO_4_; (**B**) the band gap energies of (a) g-C_3_N_4_, (b) g-C_3_N_4_/Ag_2_CrO_4_(23.1%), and (c) Ag_2_CrO_4_.

It was believed that remarkable absorption enhancement in the visible-light region was beneficial for improving photocatalytic activity. Optical absorption spectra of the prepared samples were provided by UV-vis DRS and the results in the range of 200–800 nm were shown in Fig. 5A. As can be seen, the absorption edge of the pure g-C_3_N_4_ and Ag_2_CrO_4_ located at approximately 454 nm and 712 nm, respectively. It was noteworthy that the individual Ag_2_CrO_4_ showed the photo-absorption property in the entire waveband, which suggested that an effective utilization of the solar source over Ag_2_CrO_4_ could be acquired. Interestingly, the absorption of composites was gradually strengthened in the visible region with increasing of Ag_2_CrO_4_ content. This phenomenon could be attributed to the difference between the band gap energies of bare g-C_3_N_4_ and Ag_2_CrO_4_. With more and more Ag_2_CrO_4_ nanoparticles with relative narrow band gap produced on the surface of g-C_3_N_4_, the band gap of the g-C_3_N_4_/Ag_2_CrO_4_ would decrease. Other groups also found the same trend when coupling the broader band gap semiconductors with other relatively narrow band gap semiconductors^42,65^. Therefore, introducing Ag_2_CrO_4_ into g-C_3_N_4_ might be favorable for photocatalytic reaction due to the enhancing of light absorbance. These results implied that the g-C_3_N_4_/Ag_2_CrO_4_ nanocomposite had the potential to be efficient visible light-driven photocatalyst. The UV-vis DRS spectra were also applied to calculate band gap energy (*E*_*g*_) using Tauc’s equation:

1 $$\alpha h\nu ={\rm{A}}{(h\nu -{E}_{g})}^{n/2}$$

In this equation, *α*, *hν*, and A were absorption coefficient, the photon energy, and proportionality constant, respectively^66^. The value of n was determined by the type of optical transition of a semiconductor (n = 1 for direct transition, and n = 4 for indirect transition), and the n values of g-C_3_N_4_ and Ag_2_CrO_4_ were 1^49,67^. So according to the calculation ((*αhν*)^2^ = A^2^*hν* − A^2^*E*_*g*_), the plot of (*αhν*)^2^ versus photon energy (*hν*) for g-C_3_N_4_, pure Ag_2_CrO_4_ and the as-prepared g-C_3_N_4_/Ag_2_CrO_4_(23.1%) composites were shown as Fig. 5B. The band gap energies of g-C_3_N_4_ and Ag_2_CrO_4_ were 2.73 eV and 1.74 eV, respectively. And the wavelength thresholds of the g-C_3_N_4_/Ag_2_CrO_4_(23.1%) composite were estimated at 461 and 708 nm, corresponding to the band gaps at 2.69 and 1.75 eV, respectively ascribed to g-C_3_N_4_ and Ag_2_CrO_4_. The reduced band gaps of the g-C_3_N_4_/Ag_2_CrO_4_(23.1%) composite and more response to the visible light were caused by the loaded Ag_2_CrO_4_ nanoparticles, thus more efficient utilization of solar energy could be achieved, and the improved photocatalytic activity of the g-C_3_N_4_/Ag_2_CrO_4_ composite could be anticipated.

### Visible light photocatalytic activities and evaluation of stability

**Figure 6:**
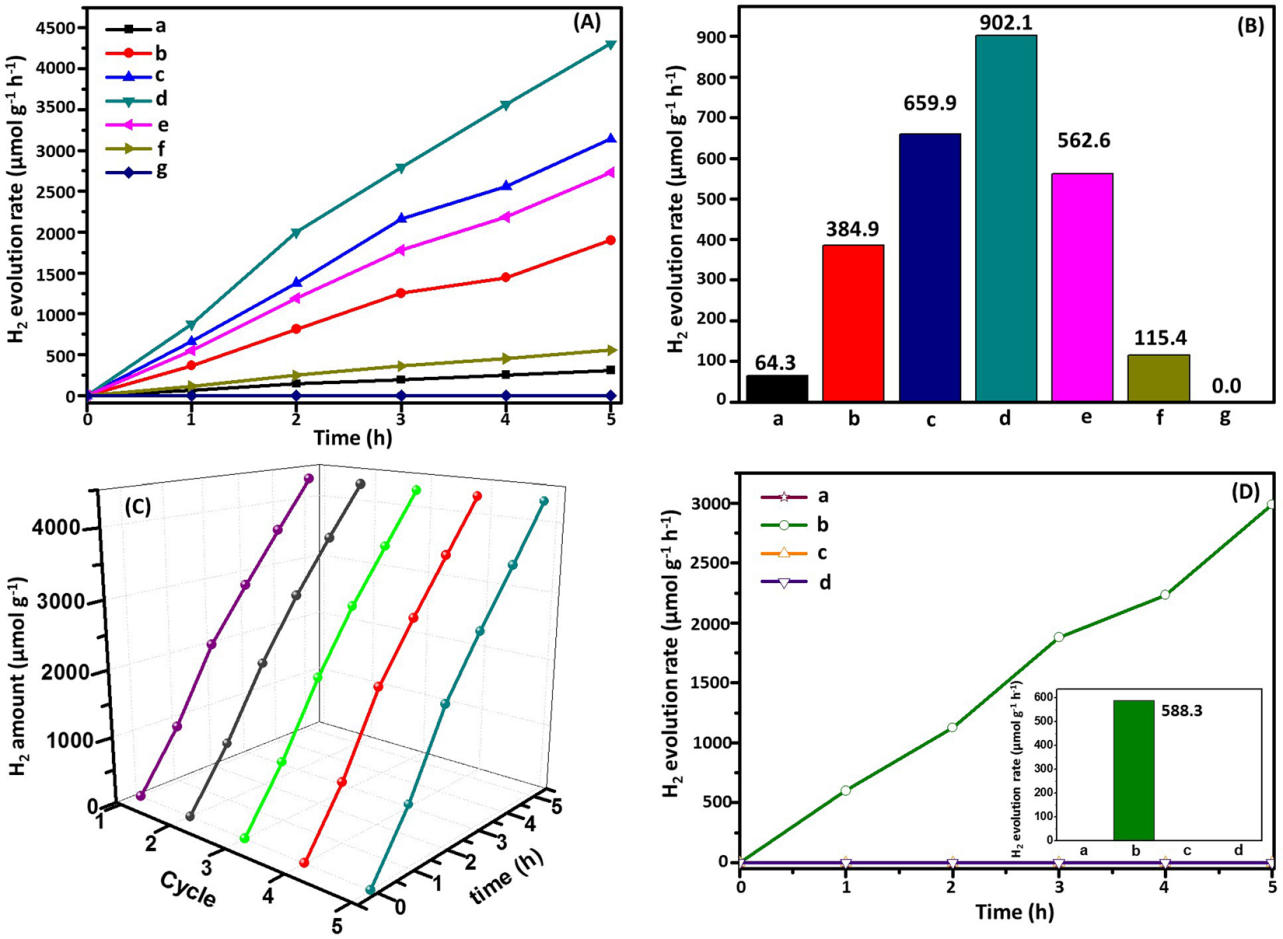
(**A**) Time courses of photocatalytic H_2_ (**B**) the average rate of H_2_ of (a) g-C_3_N_4_, (b) g-C_3_N_4_/Ag_2_CrO_4_(9.1%), (c) g-C_3_N_4_/Ag_2_CrO_4_(16.7%), (d) g-C_3_N_4_/Ag_2_CrO_4_(23.1%), (e) g-C_3_N_4_/Ag_2_CrO_4_(28.6%), (f) g-C_3_N_4_/Ag_2_CrO_4_(33.3%) and (g) Ag_2_CrO_4_ under visible light irradiation; (**C**) Recyclability of the g-C_3_N_4_/Ag_2_CrO_4_(23.1%) in five successive experiments for the H_2_ evolution under visible light irradiation; (**D**) Time courses of photocatalytic H_2_ (insert: the average rate of H_2_) of (a) g-C_3_N_4_ with methanol without Pt, (b) g-C_3_N_4_/Ag_2_CrO_4_(23.1%) with methanol without Pt, (c) g-C_3_N_4_ without methanol with Pt, and (d) g-C_3_N_4_/Ag_2_CrO_4_(23.1%) without methanol with Pt.

The photocatalytic activity of all samples was evaluated by monitoring the hydrogen evolution in the presence of a sacrificial reagent (methanol) under visible light illumination (λ ≥ 420 nm). Control experiments indicated that there was no appreciable H_2_ production in the absence of photocatalysts, light irradiation or H_2_O. According to the standard curve: y = 165550x, R^2^ = 0.9997 (as shown in Fig. S5), time-dependent (Fig. 6A) and average (Fig. 6B) photoinduced H_2_ evolution for different photocatalysts were observed and corresponding results were listed in Table S1. As illustrated, H_2_ production was not detected if pure Ag_2_CrO_4_ was used as a photocatalyst for the whole visible-light irradiation for 5 h; this suggested that Ag_2_CrO_4_ alone was not active for photocatalytic H_2_ generation. Pure g-C_3_N_4_ showed a poor photocatalytic activity under visible light (64.3 μmol g^−1^ h^−1^), owing to its limited light-harvesting efficiency and fast recombination of photogenerated electron-hole pairs. However, after the two semiconductors were combined, the evolution rate of g-C_3_N_4_/Ag_2_CrO_4_ was greatly enhanced and the largest rate as high as 902.1 μmol g^−1^ h^−1^ was attained by using g-C_3_N_4_/Ag_2_CrO_4_(23.1%) as photocatalyst, which was 14 times that of g-C_3_N_4_. Compared with other g-C_3_N_4_ based composite photocatalysts, the H_2_ production rate of g-C_3_N_4_/Ag_2_CrO_4_ was enhanced to a great extent, which could be ascribed to the effect of Ag_2_CrO_4_ (Table 1). In the scope of our literature survey, our efficiency of hydrogen production is very high. It is just lower compared to that of literature ref.^6^ and ref.^15^. Furthermore, it could also be clearly observed that the content of Ag_2_CrO_4_ exhibited a great influence on the H_2_ production rate. With its increasing, the H_2_ production rate rose firstly and then decreased. For bare g-C_3_N4, the H_2_ production rate was extremely low because of the deeply trapped electrons and fast recombination of electron-hole pairs; the photogenerated electrons were not transferred to catalytic sites and were unable to participate in H_2_ production^68^. As a compare, the pure g-C_3_N_4_ and g-C_3_N_4_/Ag_2_CrO_4_(23.1%) was conducted photogenerated hydrogen without Pt co-catalyst or methanol as electro donors. From Fig. 6D (the corresponding results were listed in Table S3), we could see that with methanol but not Pt, there was no observable hydrogen generation be detected using pure g-C_3_N_4_ as photocatalyst under visible light irradiation. And the hydrogen production efficiency of g-C_3_N_4_/Ag_2_CrO_4_(23.1%) was reduced. While without any electron donors such as methanol, no H_2_ is produced either using pure g-C_3_N_4_ or g-C_3_N_4_/Ag_2_CrO_4_(23.1%) as a catalyst.

**Table 1:** Comparison of the photocatalytic H_2_ production rate reported in the literatures with Z-scheme g-C_3_N_4_/Ag_2_CrO_4_ in our work with methanol as sacrificial agent under visible light irradiation.

Sample	Efficiency (μmol h^−1^ g^−1^)	Co-catalyst (Pt)	Light source	Refrence
g-C_3_N_4_/InVO_4_	212	0.6%	>420 nm	ref.^5^
g-C_3_N_4_/NiFe-LDH	24800	No	≥420 nm	ref.^6^
GCN/NT NFs	8931.3	A certain amount	simulated solar light	ref.^15^
CdS/Au/g-C_3_N_4_	19	No	>420 nm	ref.^16^
Fe_2_(MoO_4_)_3_/g-C_3_N_4_	0.18	No	>420 nm	ref.^17^
Au/SnO_2_/g-C_3_N_4_	770	No	>400 nm	ref.^21^
Au/PtO/g-C_3_N_4_	16.9	No	>400 nm	182^22^
TiO_2_/g-C_3_N_4_	74.7	0.5%	>400 nm	ref.^23^
MoS_2_/g-C_3_N_4_	231	1%	>400 nm	ref.^24^
g-C_3_N_4_/Au/P25	259	No	simulated solar light	ref.^25^
Fe/P-g-C_3_N_4_	150.6	No	>400 nm	ref.^26^
g-C_3_N_4_/WS_2_	101	No	≥420 nm	ref.^27^
Ag_2_S/g-C_3_N_4_	200	No	=420 nm	ref.^28^
g-C_3_N_4_/TiO_2_	559.7	No	Full light	ref.^29^
g-C_3_N_4_/Ag_2_CrO_4_	902.1	0.6%	≥420 nm	Our work

To evaluate the stability and reusability of the g-C_3_N_4_/Ag_2_CrO_4_ nanocomposites, a recycling test of the g-C_3_N_4_/Ag_2_CrO_4_(23.1%) (5 h) was performed and the corresponding results were displayed in Fig. 6C and the corresponding results were listed in Table S2. The amount of H_2_ produced increased steadily with an extension in the reaction time and no significant deactivation was observed after 5 cycles, indicating that the g-C_3_N_4_/Ag_2_CrO_4_ nanocomposites had high stability in the photocatalytic H_2_ evolution. Furthermore, the XRD of g-C_3_N_4_/Ag_2_CrO_4_(23.1%) before and after H_2_ production were shown as Fig. S6. It was seen that the peaks of the catalyst were similar, and the morphology was steady. Furthermore, there were no new peaks according to Pt appeared due to its low content. And the TEM of g-C_3_N_4_/Ag_2_CrO_4_(23.1%) after five cycles also indicated that Ag_2_CrO_4_ nanoparticless were still anchored on the surface of g-C_3_N_4_ (Fig. S7), suggesting a good stability of inherent structure for g-C_3_N_4_ and Ag_2_CrO_4_ nanoparticles. Such high stability might result from the formation of the heterostructure between g-C_3_N_4_ and Ag_2_CrO_4_.

### Charge transfer properties

**Figure 7:**
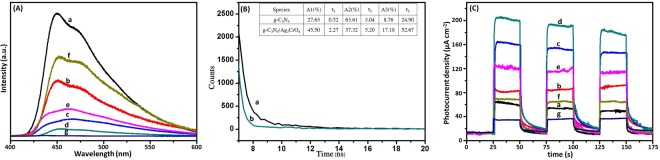
(**A**) Photoluminescence spectra of (a) g-C_3_N_4_, (b) g-C_3_N_4_/Ag_2_CrO_4_(9.1%), (c) g-C_3_N_4_/Ag_2_CrO_4_(16.7%), (d) g-C_3_N_4_/Ag_2_CrO_4_(23.1%), (e) g-C_3_N_4_/Ag_2_CrO_4_(28.6%), (f) g-C_3_N_4_/Ag_2_CrO_4_(33.3%) and (g) Ag_2_CrO_4_; (**B**) Time-resolved photoluminescence spectra for (a) g-C_3_N_4_, (b) g-C_3_N_4_/Ag_2_CrO_4_(23.1%); (**C**) it of (a) g-C_3_N_4_, (b) g-C_3_N_4_/Ag_2_CrO_4_(9.1%), (c) g-C_3_N_4_/Ag_2_CrO_4_(16.7%), (d) g-C_3_N_4_/Ag_2_CrO_4_(23.1%), (e) g-C_3_N_4_/Ag_2_CrO_4_(28.6%), (f) g-C_3_N_4_/Ag_2_CrO_4_(33.3%) and (g) Ag_2_CrO_4_.

As shown in Fig. 6, the Z-scheme nanocomposite formed between g-C_3_N_4_ and Ag_2_CrO_4_ dramatically enhanced the photocatalytic performance under visible-light irradiation. To explore the effect of silver chromate on separation efficiency of the photogenerated electron-hole pairs on the g-C_3_N_4_, PL spectra for the g-C_3_N_4_, g-C_3_N_4_/Ag_2_CrO_4_ and Ag_2_CrO_4_ samples were provided in the range of 400–600 nm and the results were displayed in Fig. 7A. Since PL emission spectra derived from the recombination of free carriers, it was generally accepted that the PL spectrum with low intensity indicated efficiently separation of the charge carriers, leading to participation of more electrons and holes in the oxidation and reduction reactions. It could be observed that all of the samples had similar spectra in this range. It was clear to see that the bare g-C_3_N_4_ had a wide and strong peak around 450 nm. However, there was a considerable decrease in the intensity of the PL spectrum for the g-C_3_N_4_/Ag_2_CrO_4_ nanocomposites compared to that of the pure g-C_3_N_4_. Hence, the photogenerated electron-hole pairs could migrate easily between g-C_3_N_4_ and Ag_2_CrO_4_, due to the matching band potentials^30,69^. It was noted that the g-C_3_N_4_/Ag_2_CrO_4_(23.1%) sample showed the weakest emission intensity, meaning that it had the lowest recombination rate of the photogenerated charge carriers and the highest photocatalytic activity compared with other composites. Additionally, it was worth noting that the peaks density of PL first declined, then increased along with the further increasing content of Ag_2_CrO_4_. That was excess Ag_2_CrO_4_ species increased the recombination rate of photogenerated charge carriers, leading to inferior photocatalytic efficiency, arising from the excess Ag_2_CrO_4_ species might agglomerate seriously leading to decrease of the combinations between counterparts of the nanocomposite and acted as a recombination center and thereby reducing the efficiency of charge separation^70–72^. The time-resolved photoluminescence spectra for g-C_3_N_4_ and g-C_3_N_4_/Ag_2_CrO_4_(23.1%) were tested and shown in Fig. 7B. It could be clearly observed that g-C_3_N_4_/Ag_2_CrO_4_(23.1%) showed longer decay time value compared to pure g-C_3_N_4_ which could be ascribed to the effective charge transfer across the interface of g-C_3_N_4_ and Ag_2_CrO_4_^73^.

Consequently, we recorded the transient photocurrent responses of the pure g-C_3_N_4_, g-C_3_N_4_/Ag_2_CrO_4_ nanocomposite and Ag_2_CrO_4_ under dark conditions and visible light irradiation. Fig. 7C showed a comparison of the photocurrent-time curves for photocatalysts, with three on-off intermittent irradiation cycles. It was widely considered as the most efficient evidence for explaining the electrons and holes separation in the composite photocatalysts^38,74,75^. Generally, the corresponding relationship was recognized as follows: the higher photocurrent implied the higher electrons-holes separation efficiency, thus leading to the higher photocatalytic activity. It was clear that the photocurrent densities rapidly decreased to zero as soon as the lamp was turned off, and that the photocurrent densities maintained stable values when the lamp was turned on, indicating a rapid photocurrent response to the on-off intermittent irradiation. It could be obviously seen that the photocurrent responses of the g-C_3_N_4_/Ag_2_CrO_4_ nanocomposites were increased significantly in comparison with that of pure g-C_3_N_4_, indicating a higher separation and transfer efficiency of the photogenerated electron-hole pairs under visible light irradiation, and hence higher photocatalytic activity. Additionally, it was worth noting that the photocurrent density first increased, then declined along with the increasing content of Ag_2_CrO_4_, which was in well accordance with the above PL results. This provided further evidence to support the above PL results. Hence, the abovementioned results obviously confirmed the superior charge transfer and recombination inhibition in the g-C_3_N_4_/Ag_2_CrO_4_ composites in comparison with only g-C_3_N_4_, demonstrating that the introduction of Ag_2_CrO_4_ could effectively enhance the separation and transfer efficiency of photogenerated electron-hole pairs of g-C_3_N_4_, which could improve the photocatalytic performance.

### Mechanism of enhanced photoactivity

**Figure 8:**
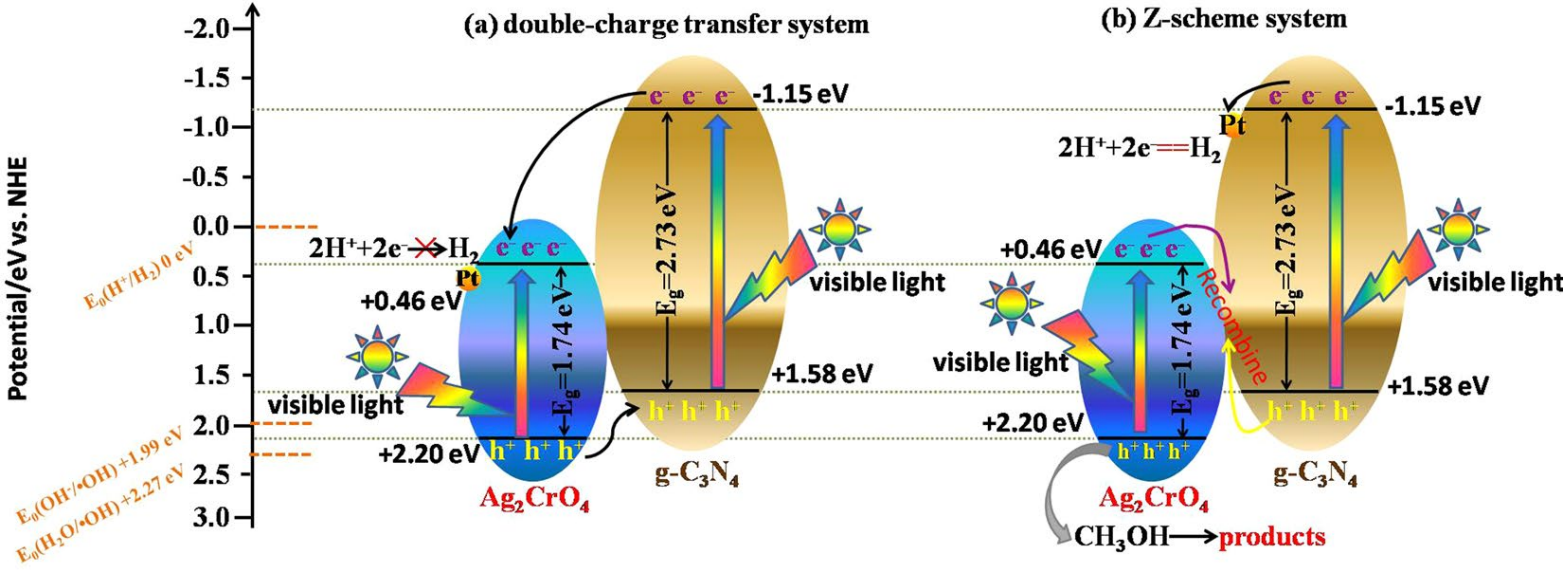
Schematic representation of the charge generation, migration and hydrogen production mechanism of Z-scheme g-C_3_N_4_/Ag_2_CrO_4_ nanocomposites.

As far as bare g-C_3_N_4_ was concerned, normally, the photogenerated electron-hole pairs quickly recombined and only a fraction of them participated in water splitting reaction, resulting in low photocatalytic activity^5,6,15,21^. Based on the above analysis of the experiment and characterization results, the possible photocatalytic reaction mechanism and electron transfer processes of the g-C_3_N_4_/Ag_2_CrO_4_ nanocomposites were proposed (Fig. 8). The conduction band potential (*E*_*CB*_) and valence band potential (*E*_*VB*_) of the semiconductors could be calculated by the following empirical equations^76^:

2 $${E}_{CB}=X-{E}^{e}-\frac{1}{2}{E}_{g}$$

3 $${E}_{VB}={E}_{CB}+{E}_{g}$$

where *X* was the absolute electronegativity of the semiconductor, which is the geometric mean of the electronegativity of the constituent atoms, and the values of the *X* for g-C_3_N_4_ and Ag_2_CrO_4 _are 4.72 eV and 5.83 eV, respectively^52,77^. *E*^*e*^ was the energy of free electrons on the hydrogen scale (∼4.50 eV vs NHE). Therefore, the *E*_*CB*_ potentials of g-C_3_N_4_ and Ag_2_CrO_4_ were −1.15 eV and +0.46 eV, and corresponding *E*_*VB*_ potentials could be estimated to be +1.58 eV and +2.20 eV, respectively. The values of VB for g-C_3_N_4_ and Ag_2_CrO_4_ were also obtained by XPS and the results for them were 1.60 eV and 2.20 eV respectively (shown as Fig. S3), which were similar with the results of calculation ones. Under visible light irradiation, Ag_2_CrO_4_ and g-C_3_N_4_ were both excited and yield electron-hole pairs. According to the double-charge transfer theory (as shown in Fig. 8a)^78–80^, the photo-generated electrons of g-C_3_N_4_ would migrate to the conduction band (CB) of Ag_2_CrO_4_ and focused on the Pt nanoparticales, then the holes of Ag_2_CrO_4_ would transfer to the valence band (VB) of g-C_3_N_4_, because the VB and CB potentials of Ag_2_CrO_4_ were both lower than those of g-C_3_N_4_. However, if the composites followed the double charge transfer theory, the electrons in the CB of Ag_2_CrO_4_ could not reduce H^+^ to generate H• due to the CB potential of Ag_2_CrO_4_ (0.46 eV/vs. NHE) was higher than E_0_ (H^+^/H_2_) (0 eV/vs. NHE). Therefore, a Z-scheme mechanism was proposed based on the above analysis. As illustrated in Fig. 8b, the photo-generated electrons on the CB of Ag_2_CrO_4_ could easily transfer to the VB of g-C_3_N_4_ and reacted with the holes of g-C_3_N_4_, effectively inhibiting the electron-hole pairs recombination in both Ag_2_CrO_4_ and g-C_3_N_4_, prolonging the lifetime of the photogenerated electrons on the CB of g-C_3_N_4_ and the photogenerated holes on the VB of Ag_2_CrO_4_, so enhancing the interfacial charge transfer. Furthermore, Pt as co-catalyst could accept and transfer electrons and functioning as an effective hydrogen evolution promoter for g-C_3_N_4_. In such a way, the photogenerated electrons and holes were efficiently separated by the Z-scheme charge transfer; it could be further confirmed by the PL spectra and photocurrent analysis. Subsequently, the photogenerated electrons left at the CB of g-C_3_N_4_ which have more negative potential than the standard redox potential of H^+^/H_2_ (0 eV vs. NHE) could reduce H^+^ to yield H• which was the source to produce hydrogen. Holes stored in the VB of Ag_2_CrO_4_ could directly react with sacrificial agent (methanol).

## Conclusion

In summary, an efficient visible-light-driven bio-inspired Z-scheme g-C_3_N_4_/Ag_2_CrO_4_ heterostructure nanocomposite had been successfully fabricated via a facile chemical precipitation method and was applied in the photocatalytic H_2_ generation. The obtained g-C_3_N_4_/Ag_2_CrO_4_ photocatalysts exhibited excellent hydrogen evolution efficiency in comparing with the individual Ag_2_CrO_4_ and g-C_3_N_4_ under visible light irradiation (λ ≥ 420 nm). The composite with the optimal mass ratio of 23.1% exhibited the highest photocatalytic activity, which could be achieved 902.1 μmol g^−1^ h^−1^. The enhanced photocatalytic activityis ascribed to the formation of the Z-scheme g-C_3_N_4_/Ag_2_CrO_4_ heterostructures which possessed higher separation and transfer efficiencies of the photogenerated electron-hole pairs. Moreover, owing to the firmly combination between Ag_2_CrO_4_ and g-C_3_N_4_ in heterostructures, the photocorrosion of Ag_2_CrO_4_ nanoparticles was strongly suppressed. This study might help to understand the mechanism of the g-C_3_N_4_/silver composites and provided a new insight to the design of the Z-scheme heterostructures.

## Experimental Section

### Preparation

All reagents (except the urea was chemical grade) were analytical grade and used without further purification. The product of bulk g-C_3_N_4_ was obtained by a simple calcination method^81^. Typically, the precursor urea (10 g) was calcined at 600 °C for 4 h with a ramp rate of 5 °C/min in a covered alumina crucible in order to prevent sublimation of urea and kept the calcinations took place in a static air atmosphere. The obtained light-yellow powder was washed and dried at 50 °C in a vacuum oven. After that the light-yellow products were milled into powder in an agate mortar for further experiments.

The g-C_3_N_4_/Ag_2_CrO_4_ composites were prepared by an *in-situ* chemical precipitation method under room temperature. Typically, 100 mg as-prepared g-C_3_N_4_ samples were immersed into 40 mL ultra-pure water and ultrasonically dispersed for 15 min. After that, 17.6 mg of K_2_CrO_4_ was added to the suspension under stirring at room temperature for 1 h, and then was treated using ultrasonic treatment for 1 h to ensure that CrO_4_^2−^ was fully adsorbed on the surface of g-C_3_N_4_ sheets. Afterwards, an aqueous solution of AgNO_3_ (30.8 mg in 20 mL of water) was drop wise added to the suspension under stirring. After stirring for another 2 h, the precipitate was collected by centrifugation and washed with ethanol and water for several times, and dried at 60 °C for 12 h under vacuum condition. Finally, the g-C_3_N_4_/Ag_2_CrO_4_ composite with a theoretical weight ratio of Ag_2_CrO_4_ to g-C_3_N_4_ at 30:100 was prepared and named as g-C_3_N_4_/Ag_2_CrO_4_(23.1%). The g-C_3_N_4_/Ag_2_CrO_4_ composites with different mass ratios were fabricated by changing the addition amount of AgNO_3_ and K_2_CrO_4_ solution. The samples were marked as g-C_3_N_4_/Ag_2_CrO_4_(9.1%), g-C_3_N_4_/Ag_2_CrO_4_(16.7%), g-C_3_N_4_/Ag_2_CrO_4_(28.6%), and g-C_3_N_4_/Ag_2_CrO_4_(33.3%). The Ag_2_CrO_4_ was synthesized by the similar method but without g-C_3_N_4_.

### Characterization

The phase structures were analyzed by X-ray diffraction (XRD) with a powder diffractometer (XRD-6000, Shimadzu, Japan). The topographies were observed by using transmission electron microscopy (TEM, Tecnai G^2^ 20 S-TWIN, FEI, America). Energy dispersive spectroscopy (EDS) was obtained from a scanning electron microscopy (JSM 7500F, Japan Electron Optics Laboratory Co., Ltd., Japan) equipped with EDS attachment (INCA Energy 250, Oxford, America). Chemical compositions of the samples were also analyzed using X-ray photoelectron spectroscopy (XPS, ESCALAB 250Xi, Thermo Fisher, America). Uv-visible diffuse reflectance spectra (UV-vis DRS) were collected on a spectrophotometer (UV-3600, Shimadzu, Japan). Fourier-transform infrared (FT-IR) spectra were obtained on a FT-IR spectrophotometer (Nicolet iS10 IR, Thermo Scientific, America). Photoluminescence (PL) spectra were recorded on a fluorescence spectrometer (F-4500, Hitachi, Japan) with photomultiplier tube voltage of 400 V and scanning speed of 240 nm/min. The photocurrent experiments were performed on an electrochemical workstation (CHI 660D, Shanghai Chen Hua Instrument Co., Ltd., China) in a standard three-electrode system with Pt plate and Ag/AgCl (saturated KCl) electrode as counter electrode and reference electrode, respectively. A solar simulator illumination (CXE-350, Beijing Aodite Photoelectronic Technology Co., Ltd., China) at intensity of 100 mW cm^−2^ was used. The working electrode was prepared as follows: 10 mg powder was dispersed ultrasonically in 0.5 mL of mixture solution (water: ethanol: Nafion (5%) = 230:250:20). Then, 20 μL of the resulting colloidal dispersion (20 mg/mL) was drop-cast onto a piece of ITO with a fixed area of 1 cm × 1 cm, and the electrodes were dried under ambient for 4 h.

### Photocatalytic activity of H_2_ evolution

Photocatalytic H_2_-evolution experiments were performed in a gas-closed circulation system (Labsolar-6A, Beijing Perfectlight Technology Co., Ltd., China) with a top-irradiation quartz vessel. In a typical experiment, 50 mg of the as-prepared photocatalyst was dispersed in 100 mL mixed solution containing 75 mL water and 25 mL methanol (with 10 mM NaHCO_3_). 0.6% of co-catalyst Pt was deposited onto the surface of the photocatalyst by in-site photo-deposition when a certain amount of H_2_PtCl_6_ solution was added. Before irradiation, the air in the system was removed by a vacuum pump. A 300 W Xe lamp (light intensity: 200 mW cm^−2^, Microsolar300, Beijing Perfectlight Technology Co., Ltd., China) with a 420 nm cut off filter was used as the visible light source. The produced hydrogen was *in situ* detected periodically using an online gas chromatograph (GC7900, Tech-comp Shanghai Co., Ltd., China) with argon as carried gas.

## Electronic supplementary material

Supplementary Information
