# Supplementary material for: Bio-inspired Z-scheme g-C3N4/Ag2CrO4 for efficient visible-light photocatalytic hydrogen generation
Source: Sci Rep. 2018 Nov 7;8:16504. doi: 10.1038/s41598-018-34287-w (PMC6220202; doi:10.1038/s41598-018-34287-w)
Supplement: Supplementary file 1 — Supplementary Information [file 41598_2018_34287_MOESM1_ESM.docx]

**Bio-inspired** **Z-scheme** **g-C_3_N_4_/Ag_2_CrO_4_** **for efficient visible-light photocatalytic** **hydrogen generation**

Yuping Che^1^, Bingxin Lu^1^, Qi Qi^1^, Huaiqiu Chang^3^, Jin Zhai^1,*^ Kefeng Wang^2,*^ & Zhaoyue Liu^1^

^1^Key Laboratory of Bio-Inspired Smart Interfacial Science, Technology of Ministry of Education and Beijing Advanced Innovation Center for Biomedical Engineering, Beijing Key Laboratory of Bio-inspired Energy Materials and Devices, School of Chemistry, Beihang University, Beijing 100191, P. R. China

^2^Henan Key Laboratory of Biomolecular Recognition and Sensing, Shangqiu Normal University, Shangqiu, 476000, P. R. China

^3^National Center for Nanoscience and Technology, Beijing 100190, P. R. China

*Corresponding Author: [**zhaijin@buaa.edu.cn**](mailto:zhaijin@buaa.edu.cn)**; wangkf2007@163.com**

**Transmission electron microscopy (TEM) analysis**


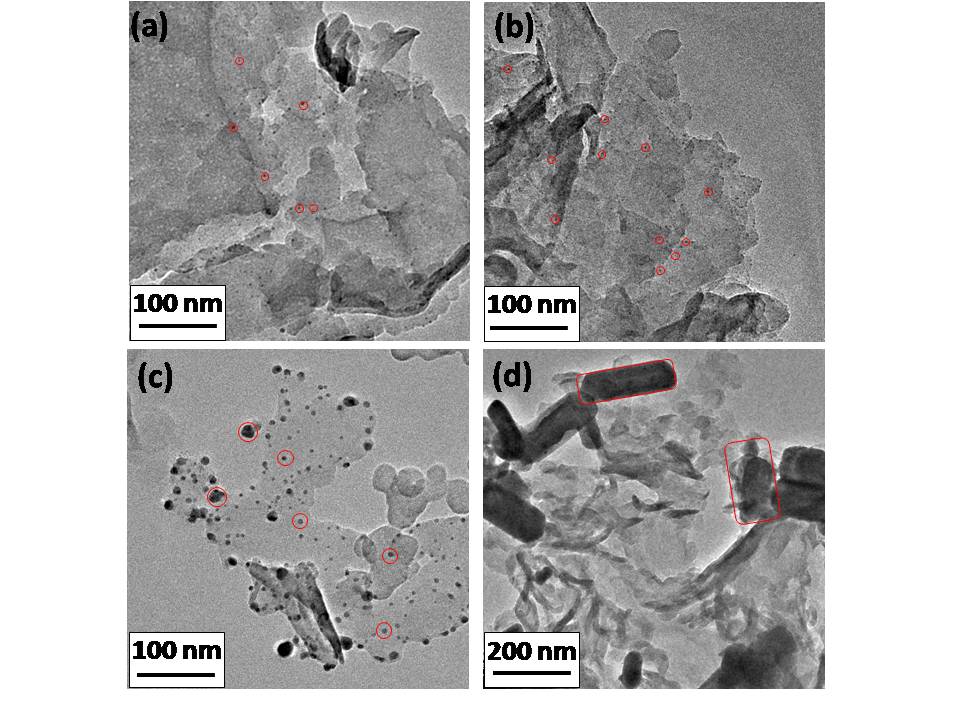


**Figure S1** TEM images of (a) Ag_2_CrO_4_/g-C_3_N_4_(9.1%), (b) Ag_2_CrO_4_/g-C_3_N_4_(16.7%), (c) Ag_2_CrO_4_/g-C_3_N_4_(28.6%) and (d) Ag_2_CrO_4_/g-C_3_N_4_(33.3%), (Ag_2_CrO_4_ were labeled by red circles).

**X-ray photoelectron spectroscopy (XPS) analysis**

To ascertain in-depth the information about the functional group and surface electronic state of g-C_3_N_4_, Ag_2_CrO_4_, and g-C_3_N_4_/Ag_2_CrO_4_(23.1%) composites, the X-ray photoelectron spectroscopy (XPS) spectra were used as shown in Figure S2. It was obviously seen that only elements of C, N, Ag, Cr and O could be found in the XPS survey spectrum (Figure S2A) of the composite, and no impurities were detectable. The corresponding high-resolution XPS spectra were shown in Figure S2B-F. Figure S2B showed the XPS spectrum of Ag 3d spectrum, the two peaks located at 367.7 eV, 368.1 eV and 373.8 eV, 374.1 eV were attributed to Ag 3d_5/2_ and Ag 3d_3/2_ of Ag_2_CrO_4_ and g-C_3_N_4_/Ag_2_CrO_4_(23.1%), respectively [^1-3^](#_ENREF_1), suggesting the presence of Ag^+^ species for Ag_2_CrO_4_. Where those at 368.5 and 374.5 eV were related to Ag0 of Ag2CrO4, the generated trace amounts of Ag0 during preparation process mainly implied the photosensitivity of Ag_2_CrO_4_[^4^](#_ENREF_4). Interestingly, the binding energy of Ag^+^ for g-C_3_N_4_/Ag_2_CrO_4_(23.1%) was higher than pure Ag_2_CrO_4_, which was much different from other elements. The blue shift of Ag^+^ peak probably revealed that an increased electron density in Ag^+^, indicating the existence of electron transfer between Ag_2_CrO_4_ and g-C_3_N_4_ nanosheets uponhybridization[^5^](#_ENREF_5). This phenomenon further confirmed that the interactions were existed in the hybrid structure, which might be conducive to migration of photo-generated charge carriers and thus enhanced the photocatalytic performance. For Cr 2p spectrum of Ag_2_CrO_4_, the two peaks located at 578.6 eV and 587.7 eV were attributed to Cr 2p_3/2_ and Cr 2p_1/2_, respectively [^6-8^](#_ENREF_6), demonstrating the presence of Cr^6+^ species for Ag_2_CrO_4_. And the corresponding peaks for the g-C_3_N_4_/Ag_2_CrO_4_(23.1%) were located at 578.8 eV and 587.9 eV. The change of the peak position were attributed to the combination of Ag_2_CrO_4_ to the g-C_3_N_4_[^9^](#_ENREF_9). The O 1s high resolution XPS spectrum (shown as Figure S2D) could be split into two peaks: the main peak at 529.9 eV was attributed to the crystal lattice oxygen of Ag2CrO4. While the weak peak at 531.8 eV and 531.4 eV were assigned to water species or external -OH group adsorbed on the surface of the sample [^6^](#_ENREF_6)^,^[^10^](#_ENREF_10). Moreover, the high-resolution X-ray photoelectron spectra (HRXPS) of C 1s shown in Figure S2E, only one peak at 284.9 eV could be observed in pure Ag_2_CrO_4_ which was related to adsorptive adventitious hydrocarbon. While for g-C_3_N_4_ and g-C_3_N_4_/Ag_2_CrO_4_(23.1%), C 1s exhibited three distinct peaks with the binding energies at 284.9 eV 286.1 eV, 288.2 eV and 284.9 eV 286.0 eV, 288.1 eV could be observed. The peak of 284.9 eV was related to adsorptive adventitious hydrocarbon. And the peaks around 286.1 eV and 286.0 eV could be contributed to sp^2^-hybridized carbon (N-C=N) in the aromatic ring. The last peaks around 288.2 eV and 288.1 eV for g-C_3_N_4_ and g-C_3_N_4_/Ag_2_CrO_4_(23.1%) were belonged to combination of C-N groups in g-C_3_N_4_. That suggested the major carbon environment in the g-C_3_N_4_ [^11^](#_ENREF_11)^,^[^12^](#_ENREF_12). Besides, the N 1s XPS spectrum shown in Figure S2F was with binding energies of 398.7, 400.1, 401.4 eV and 398.6, 400.0, 401.2 eV for g-C_3_N_4_ and g-C_3_N_4_/Ag_2_CrO_4_(23.1%) respectively. The main peak at 398.7 eV and 398.6 eV were typical signal for sp^2^-hybridized nitrogen (C-N=C) of triazine rings dominated in the g-C_3_N_4_, while the weak peaks centered at 400.1 eV, 401.4 eV and 400.0, 401.2 eV could be assigned to the tertiary N in N-(C)_3_ units and amino groups with a hydrogen atom (C-N-H), respectively ^10-11^. The peaks around 404.6 eV and 404.5 eV were caused by the positive charge localization in heterocycles [^13^](#_ENREF_13)^,^[^14^](#_ENREF_14). It could be seen that the binding energy of Ag 3d of g-C_3_N_4_/Ag_2_CrO_4_(23.1%) showed a slight positive shift in comparison with that of pure Ag_2_CrO_4_. Such a positive shift could be ascribed to the interaction between closely contacted phases of g-C_3_N_4_ and Ag_2_CrO_4_. Notably, comparing with primitive g-C_3_N4 and Ag_2_CrO_4_, binding energy of C 1s, N 1s Cr and O 1s were also all slightly decreased for g-C_3_N_4_/Ag_2_CrO_4_(23.1%). The shift of binding energy was also observed in many other g-C_3_N_4_-based composites system, which indicated that there were some interactions between Ag_2_CrO_4_ and g-C_3_N_4_[^15^](#_ENREF_15)^,^[^16^](#_ENREF_16). Consequently, all these findings were in correspondence with the above EDS analysis, distinctly confirming the coexistence of g-C_3_N_4_ and Ag_2_CrO_4_ in the as-prepared g-C_3_N_4_/Ag_2_CrO_4_ composites [^17^](#_ENREF_17).


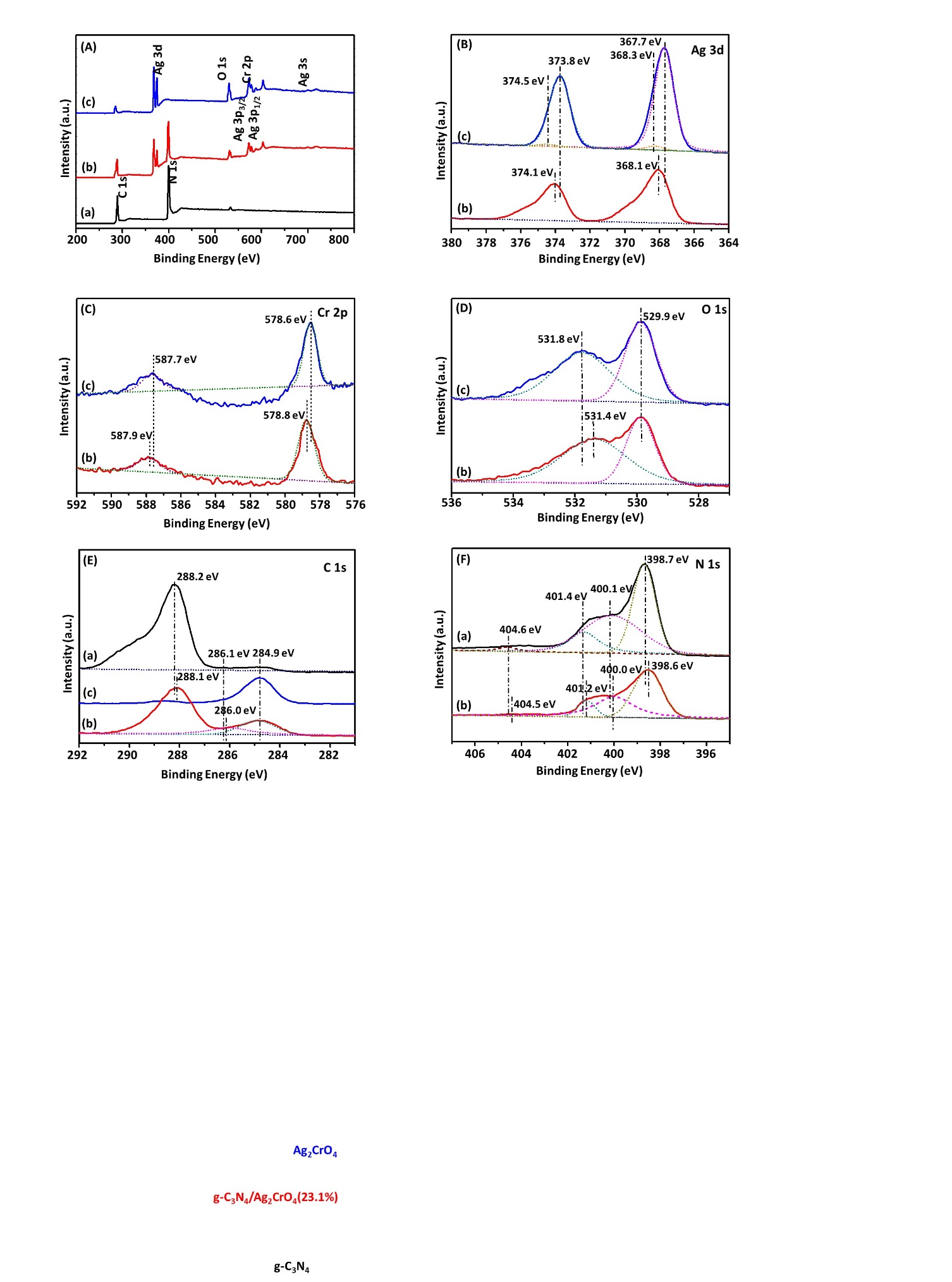


**Figure S2** (A) XPS survey spectra of composites; The corresponding high-resolution XPS spectra of composites: (B) Ag 3d (C) Cr 2p (D) O 1s, (E) C 1s, and (F) N 1s. (a) g-C_3_N_4_, (b) g-C_3_N_4_/Ag_2_CrO_4_(23.1%), (c) Ag_2_CrO_4_

**X-ray photoelectron spectroscopy of valence band (****XPS-VB) analysis**

The XPS-VB test were conducted to determine the position of the valence band and the conduction band of g-C_3_N_4_ and Ag_2_CrO_4_ (shown as Figure S3). From the test results, we could obtain that the value of VB for g-C_3_N_4_ and Ag_2_CrO_4_ were 1.60 eV and 2.20 eV respectively. For the value of conduction band, we should calculate The CB of them can be calculated using the following empirical equation:

$E_{VB}$=$E_{CB}$+$E_{g}$

*Eg* could be obtained from the results of the UV-Vis spectra. The $E_{CB}$ potentials of g-C_3_N_4_ and Ag_2_CrO_4_ were -1.13 eV and +0.46 eV.


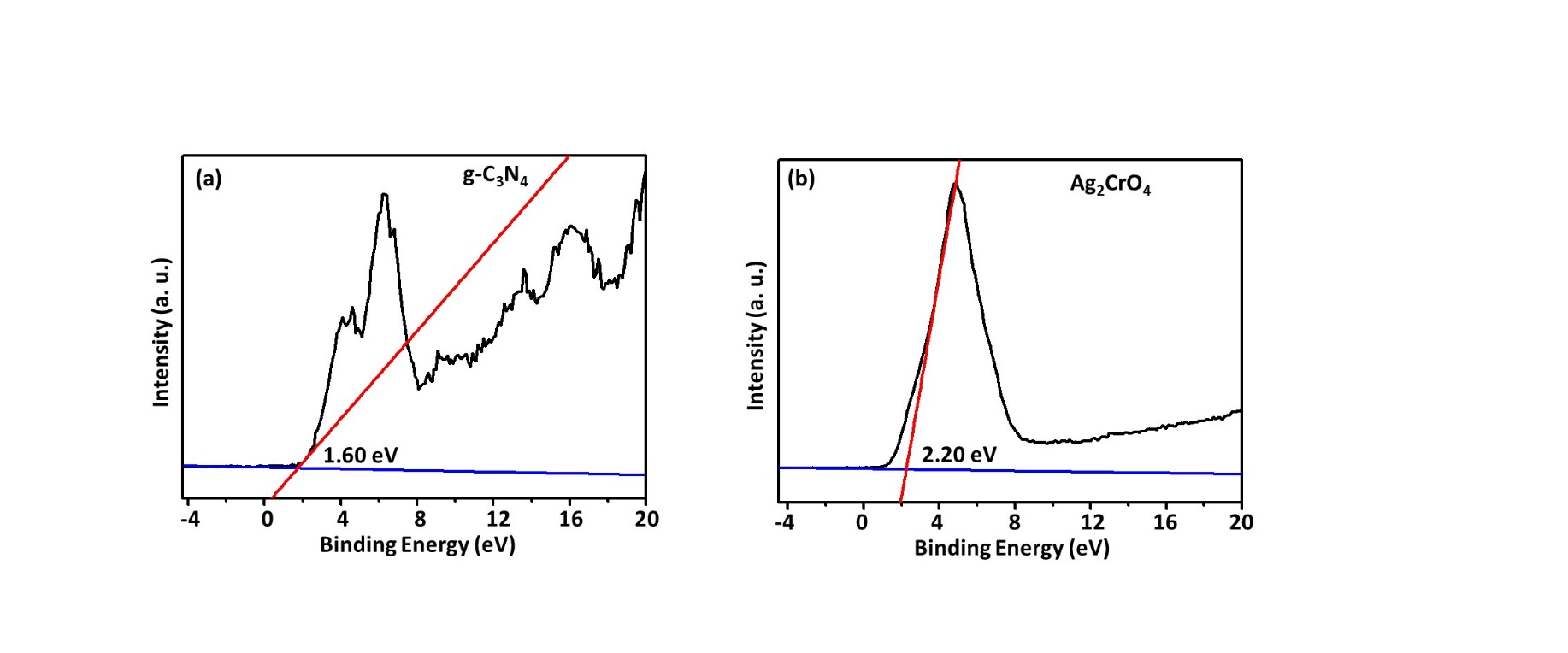


**Figure S3** XPS-VB spectra of (a) g-C_3_N_4_, (b) Ag_2_CrO_4_.

**Fourier-transform infrared (FT-IR) spectroscopy**

Infrared absorption bands were usually applied to identify components and structures of photocatalysts. Chemical structures of the g-C_3_N_4_, Ag_2_CrO_4_, and g-C_3_N_4_/Ag_2_CrO_4_(23.1%) were investigated by FT-IR spectra and the results were shown in Figure S4. All of the spectra, the peaks appeared at 3480 and 1637 cm^-1^ were indicative of hydroxyl groups stretching vibration caused by the absorption of hydrated oxide. In Figure S4a, the g-C_3_N_4_ exhibited three characteristic absorption regions located around 3200, 1200-1650, and 809 cm^-1^. The broad band at 3200 cm^-1^ was attributed to NH_2_ or N-H groups stretching vibration modes of g-C_3_N_4_ and O-H. The bands at 1240-1650 cm^-1^ were ascribed to the typical stretching modes of C-N heterocycles. The peak at 809 cm^-1^ belonged to the characteristic breathing mode of s-triazine ring units. For pure Ag_2_CrO_4_, the characteristic peak at 887 cm^-1^ with a shoulder at about 860 cm^-1^ (Figure S4c) could be assigned to the stretching vibration of the Cr-O bond in CrO_4_ tetrahedra [^18^](#_ENREF_18). All the characteristic absorption peaks of g-C_3_N_4_ and Ag_2_CrO_4_ were observed in the g-C_3_N_4_/Ag_2_CrO_4_(23.1%) (Figure S4b), revealing that Ag_2_CrO_4_ successfully composited with g-C_3_N_4_, which was consistent with XRD results.


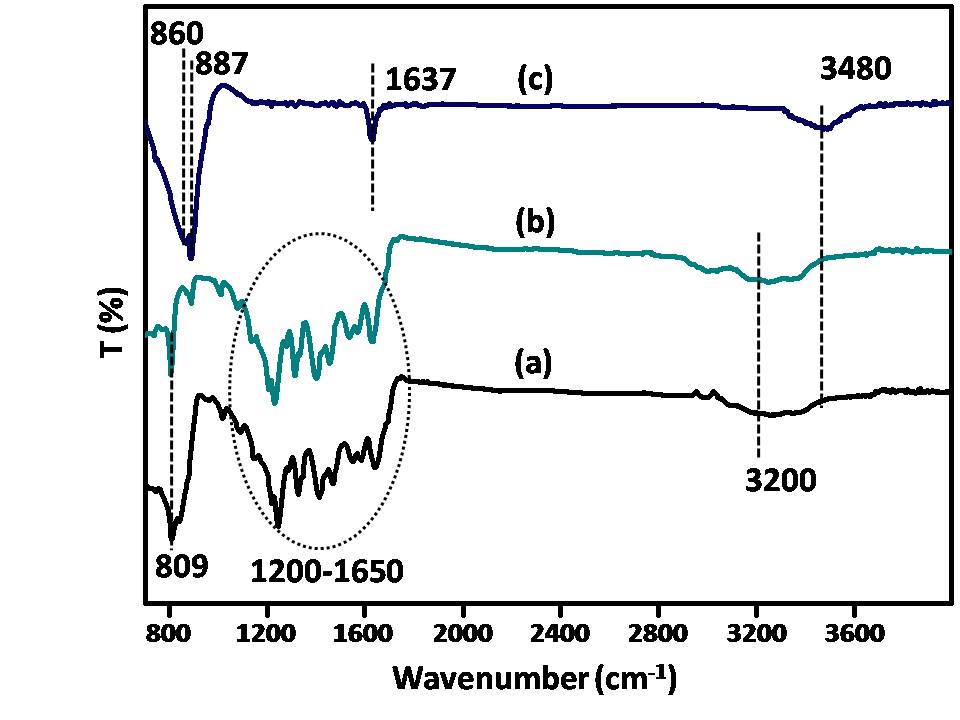


**Figure S4** FT-IR spectra of (a) g-C_3_N_4_, (b) g-C_3_N_4_/Ag_2_CrO_4_(23.1%) and (c) Ag_2_CrO_4_.

**Analysis of hydrogen generation with gas chromatographic**

The amount of hydrogen generation was determined by gas chromatography. Gas chromatographic conditions: The column was 5A (15m*3 mm*3 mm, capillary column); the inlet temperature is 120 ℃; the column temperature was 60 ℃; the TCD temperature was 120 ℃；the carrier gas is argon with a flow rate of 1 mL min^-1^; the injection mode was splitless injection; the amount of injection was 1 mL; the current was 60 mA. We injected the same amount of standard gas containing different H_2_ to get the following standard curve: y=165550x, R^2^=0.9997 (as shown in Figure S5). We used the standard curve to calculate the hydrogen generation based on the measured peak area.

**
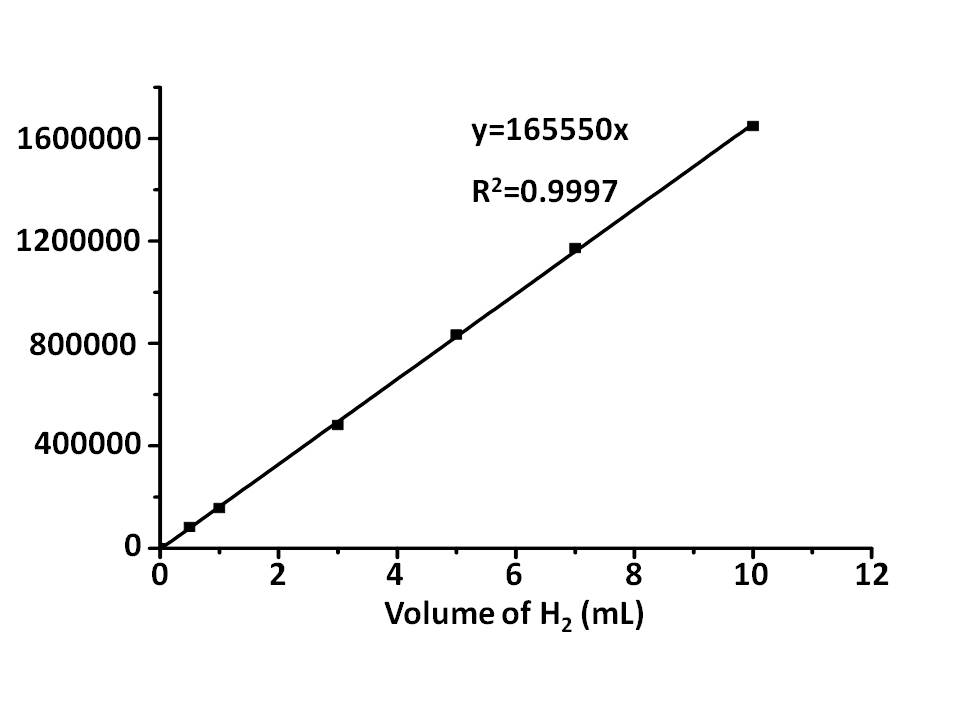
**

**Figure S5** The standard curve of the amount of hydrogen generation

**Table S1** The amount of hydrogen generation in 5 hours: (a) g-C_3_N_4_, (b) g-C_3_N_4_/Ag_2_CrO_4_(9.1%), (c) g-C_3_N_4_/Ag_2_CrO_4_(16.7%), (d) g-C_3_N_4_/Ag_2_CrO_4_(23.1%), (e) g-C_3_N_4_/Ag_2_CrO_4_(28.6%), (f) g-C_3_N_4_/Ag_2_CrO_4_(33.3%) and (g) Ag_2_CrO_4_.

| sample | 1h | | 2h | | 3h | | 4h | | 5h | |
| --- | --- | --- | --- | --- | --- | --- | --- | --- | --- | --- |
|  | Peak area (uV*s) | Amount of H_2_ (mL/μmol) | Peak area (uV*s) | Amount of H_2_ (mL/μmol) | Peak area (uV*s) | Amount of H_2_ (mL/μmol) | Peak area (uV*s) | Amount of H_2_ (mL/μmol) | Peak area (uV*s) | Amount of H_2_ (mL/μmol) |
| a | 230290 | 1.39/62.1 | 540695 | 3.27/145.8 | 718065 | 4.34/193.6 | 935635 | 5.65/252.3 | 1151850 | 6.96/310.6 |
| b | 1354670 | 8.18/365.3 | 3010556 | 18.19/811.8 | 4642443 | 28.04/1251.9 | 5349599 | 32.31/1442.6 | 7052499 | 42.60/1901.8 |
| c | 2450540 | 14.80/660.8 | 5108695 | 30.86/1377.6 | 8024175 | 48.47/2163.8 | 9481915 | 57.28/2556.9 | 11641099 | 70.32/3139.2 |
| d | 3238500 | 19.56/873.3 | 7403005 | 44.72/1996.3 | 10368210 | 62.63/2795.9 | 13209555 | 79.79/3562.1 | 15969975 | 96.47/4306.5 |
| e | 2045520 | 12.36/551.6 | 4410050 | 26.64/1189.2 | 6596399 | 39.85/1778.8 | 8102395 | 48.94/2184.9 | 10125660 | 61.16/2730.5 |
| f | 419875 | 2.54/113.2 | 920680 | 5.56/248.3 | 1333655 | 8.06/359.6 | 1675555 | 10.12/451.8 | 2064875 | 12.47/556.8 |
| g | 0 | 0/0 | 0 | 0/0 | 0 | 0/0 | 0/0 | 0/0 | 0/0 | 0/0 |

**Table S2** The stability of hydrogen generation for g-C_3_N_4_/Ag_2_CrO_4_(28.6%) in five cycles.

| Cycle | 1h | | 2h | | 3h | | 4h | | 5h | |
| --- | --- | --- | --- | --- | --- | --- | --- | --- | --- | --- |
|  | Peak area (uV*s) | Amount of H_2_ (mL/μmol) | Peak area (uV*s) | Amount of H_2_ (mL/μmol) | Peak area (uV*s) | Amount of H_2_ (mL/μmol) | Peak area (uV*s) | Amount of H_2_ (mL/μmol) | Peak area (uV*s) | Amount of H_2_ (mL/μmol) |
| 1st | 3238500 | 19.56/873.3 | 7403005 | 44.72/1996.3 | 10368210 | 62.63/2795.9 | 13209555 | 79.79/3562.1 | 15969975 | 96.47/4306.5 |
| 2nd | 3233231 | 19.53/871.9 | 7045530 | 42.56/1899.9 | 10350075 | 62.52/2791.0 | 13201435 | 79.74/3560.0 | 15960620 | 96.41/4304.0 |
| 3rd | 3234005 | 19.53/872.1 | 7045930 | 42.56/1900.0 | 10357230 | 62.56/2793.0 | 13198035 | 79.72/3599.0 | 15967975 | 96.45/4306.0 |
| 4th | 3229025 | 19.50/870.8 | 7383410 | 44.60/1991.0 | 10364920 | 62.1/2795.0 | 13186900 | 79.66/3556.0 | 15957020 | 96.39/4303.0 |
| 5th | 3222125 | 19.46/868.9 | 7390530 | 44.64/1993.0 | 10357430 | 62.56/2793.0 | 13205219 | 79.77/3561.0 | 16053350 | 96.97/4329.0 |

**Table S3** The amount of hydrogen generation in 5 hours: mixture of g-C_3_N_4_/Ag_2_CrO_4_(23.1%) with methanol but without Pt.

| 1h | | 2h | | 3h | | 4h | | 5h | |
| --- | --- | --- | --- | --- | --- | --- | --- | --- | --- |
| Peak area (uV*s) | Amount of H_2_ (mL/μmol) | Peak area (uV*s) | Amount of H_2_ (mL/μmol) | Peak area (uV*s) | Amount of H_2_ (mL/μmol) | Peak area (uV*s) | Amount of H_2_ (mL/μmol) | Peak area (uV*s) | Amount of H_2_ (mL/μmol) |
| 2221687 | 13.40/599.1 | 4174516 | 25.20/1125.7 | 6971000 | 42.08/1879.8 | 8278573 | 49.97/2232.4 | 11076170 | 66.85/2986.8 |

**Visible light photocatalytic stability**


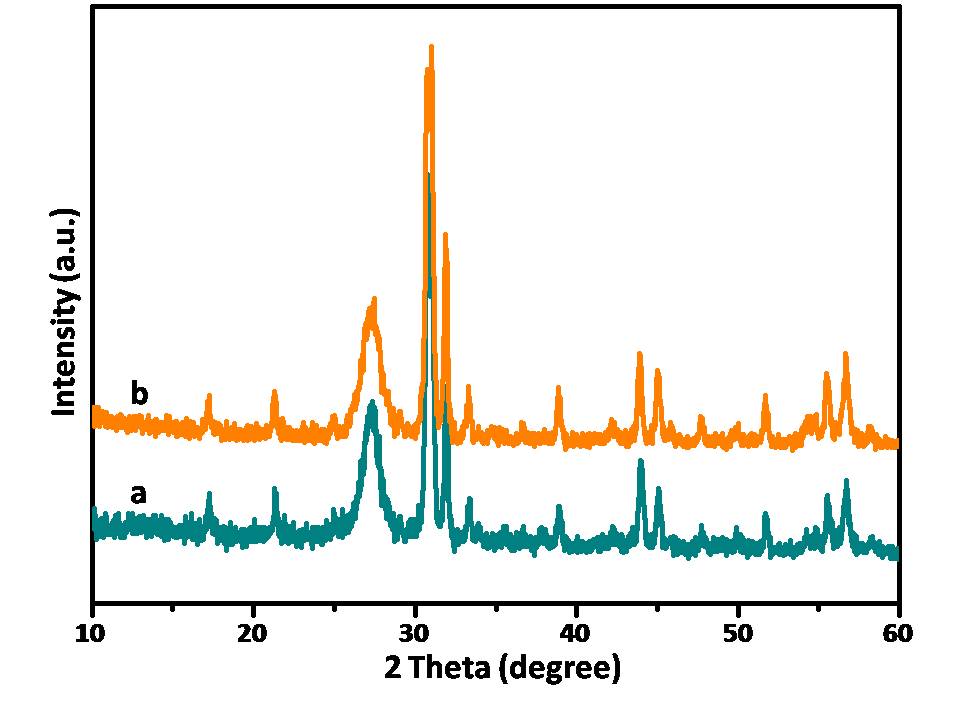


**Figure S6** XRD patterns of Ag_2_CrO_4_/g-C_3_N_4_(23.1%) (a) fresh (b) used.


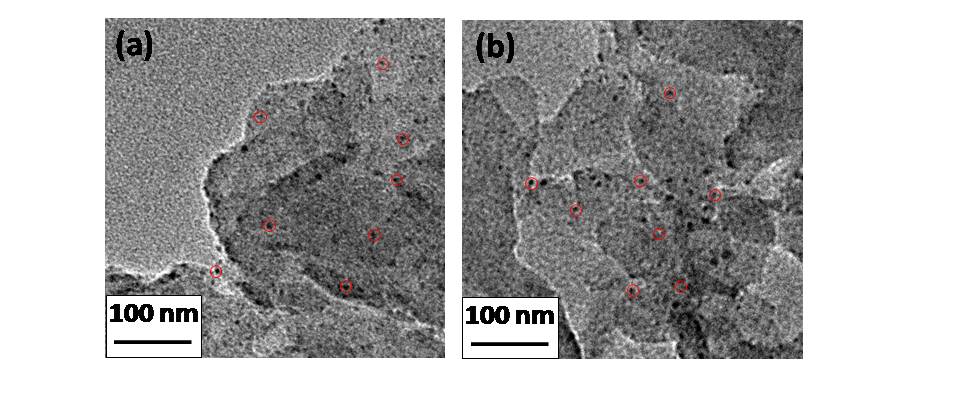


**Figure S7** TEM of Ag_2_CrO_4_/g-C_3_N_4_(23.1%) (a) fresh (b) used.

**References**

1. Guo, S. et al. Holey structured graphitic carbon nitride thin sheets with edge oxygen doping via photo-Fenton reaction with enhanced photocatalytic activity. *Appl. Catal. B-Environ.* **185**, 315-321 (2016).
2. Fan, Q. et al. Superior nanoporous graphitic carbon nitride photocatalyst coupled with CdS quantum dots for photodegradation of RhB. *Catal. Today* **264**, 250-256 (2016).
3. Yang, X. et al. Synergetic effect of MoS_2_ and g-C_3_N_4_ as cocatalysts for enhanced photocatalytic H_2_ production activity of TiO_2_. *Mater. Res. Bull.* **76**, 79-84(2016).
4. Xu, D. et al. Photocatalytic activity of Ag_2_MO_4_ (M = Cr, Mo, W) photocatalysts. *J. Mater. Chem. A* **3**, 20153-20166 (2015).
5. Zhang, S. et al. Rationally designed 1D Ag@AgVO_3_ nanowire/graphene/protonated g-C_3_N_4_ nanosheet heterojunctions for enhanced photocatalysis via electrostatic self-assembly and photochemical reduction methods. *J. Mater. Chem. A* **3**, 10119-10126 (2015).
6. Li, Z. Wu, Y. & Lu, G. Highly efficient hydrogen evolution over Co(OH)_2_ nanoparticles modified g-C_3_N_4_ co-sensitized by Eosin Y and Rose Bengal under Visible Light Irradiation. *Appl. Catal. B-Environ.* **188**, 56-64 (2016).
7. Christoforidis, K. C. et al. Synthesis and photocatalytic application of visible-light active β-Fe_2_O_3_/g-C_3_N_4_ hybrid nanocomposites. *Appl. Catal. B-Environ.* **187**, 171-180 (2016).
8. Faisal, M. et al. Synthesis of highly dispersed silver doped g-C_3_N_4_ nanocomposites with enhanced visible-light photocatalytic activity. *Mater. Design* **98**, 223-230 (2016).
9. Luo, J. et al. Synthesis and characterization of Z-scheme In_2_S_3_/Ag_2_CrO_4_ composites with an enhanced visible-light photocatalytic performance. *New J.Chem.* **41**, 845-856 (2017).
10. Hou, Y. Wen, Z. Cui, S. Feng, X. & Chen, J. Strongly coupled ternary hybrid aerogels of N-deficient porous graphitic-C_3_N_4_ nanosheets/N-doped graphene/NiFe-layered double hydroxide for solar-driven photoelectrochemical water oxidation. *Nano Lett.* **16**, 2268-2277 (2016).
11. Jian, X. et al. Construction of carbon quantum dots/proton-functionalized graphitic carbon nitride nanocomposite via electrostatic self-assembly strategy and its application. *Appl. Surf. Sci.* **370**, 514-521 (2016).
12. Chen, D. et al. Significantly enhancement of photocatalytic performances via core-shell structure of ZnO@mpg-C_3_N_4_. *Appl. Catal. B-Environ.* **147**, 554-561 (2014).
13. Qiao, F. Qi, Q. Wang, Z. Xu, K. & Ai, S. MnSe-loaded g-C_3_N_4_ nanocomposite with synergistic peroxidase-like catalysis: synthesis and application toward colorimetric biosensing of H_2_O_2_ and glucose. *Sensor Actuat. B-Chem.* **229**, 379-386 (2016).
14. Wang, Y. et al. Molten salt synthesis of water-dispersible polymeric carbon nitride nanoseaweeds and their application as luminescent probes. *Carbon* **102**, 477-486 (2016).
15. Ong, W.J. Putri, L.K. Tan, L.L. Chai, S.P. & Yong, S.T. Heterostructured AgX/g-C_3_N_4_ (X=Cl and Br) nanocomposites via a sonication-assisted deposition-precipitation approach: emerging role of halide ions in the synergistic photocatalytic reduction of carbon dioxide. *Appl. Catal. B-Environ.* **180**, 530-543 (2016).
16. Lu, M. et al. Constructing atomic layer g-C_3_N_4_-CdS nano heterojunctions with efficiently enhanced visible light photocatalytic activity. *Phy. Chem. Chem. Phy.* **16**, 21280-21288 (2014).
17. Luo, J. Zhou, X. Ma, L. & Xu, X. Rational construction of Z-scheme Ag_2_CrO_4_/g-C_3_N_4_ composites with enhanced visible-light photocatalytic activity. *Appl. Surf. Sci.* **390**, 357-367 (2016).
18. Akple, M.S. et al. Enhanced visible light photocatalytic H_2_-production of g-C_3_N_4_/WS_2_ composite heterostructures. *Appl. Surf. Sci.* **358**, 196-203 (2015).
